# North African Medicinal Plants Traditionally Used in Cancer Therapy

**DOI:** 10.3389/fphar.2017.00383

**Published:** 2017-06-26

**Authors:** Jorge M. Alves-Silva, Abderrahmane Romane, Thomas Efferth, Lígia Salgueiro

**Affiliations:** ^1^Center for Neuroscience and Cell Biology, Institute for Biomedical Imaging and Life Sciences and Faculty of Pharmacy, University of CoimbraCoimbra, Portugal; ^2^Laboratoire de Chimie Organique Appliquée, Département de Chimie, Faculté des Sciences (Semlalia), Université Cadi AyyadMarrakech, Morocco; ^3^Department of Pharmaceutical Biology, Institute of Pharmacy and Biochemistry, Johannes Gutenberg University MainzMainz, Germany

**Keywords:** anticancer, ethnobotanical, medicinal plants, North Africa, cancer

## Abstract

**Background:** Cancer is a major cause of mortality worldwide with increasing numbers by the years. In North Africa, the number of cancer patients is alarming. Also shocking is that a huge number of cancer patients only have access to traditional medicines due to several factors, e.g., economic difficulties. In fact, medicinal plants are widely used for the treatment of several pathologies, including cancer. Truthfully, herbalists and botanists in North African countries prescribe several plants for cancer treatment. Despite the popularity and the potential of medicinal plants for the treatment of cancer, scientific evidence on their anticancer effects are still scarce for most of the described plants.

**Objective:** Bearing in mind the lack of comprehensive and systematic studies, the aim of this review is to give an overview of studies, namely ethnobotanical surveys and experimental evidence of anticancer effects regarding medicinal plants used in North Africa for cancer therapy.

**Method:** The research was conducted on several popular search engines including PubMed, Science Direct, Scopus and Web of Science. The research focused primarily on English written papers published between the years 2000 and 2016.

**Results:** This review on plants traditionally used by herbalists in North Africa highlights that Morocco and Algeria are the countries with most surveys on the use of medicinal plants in folk medicine. Among the plethora of plants used, *Nigella sativa* and *Trigonella foenum-graecum* are the most referred ones by herbalists for the treatment of cancer. Moreover, a plethora of scientific evidence qualifies them as candidates for further drug development. Furthermore, we report on the underlying cellular and molecular mechanisms.

**Conclusion:** Overall, this review highlights the therapeutic potential of some medicinal plants as anticancer agents. The North African flora offers a rich source of medicinal plants for a wide array of diseases, including cancer. The elucidation of their modes of action represents an indispensable condition for the rational development of new drugs for cancer treatment. Furthermore, testing the anticancer activity *in vivo* and in clinical trials are warranted to explore the full therapeutic potential of North African plants for cancer therapy.

## Introduction

According to the World Health Organization (WHO) cancer represents a major cause for morbidity and mortality with ~14 million new cases in 2012 and 8 million cancer-related deaths (Forman and Ferlay, 2014). However, this burden is expected to even increase to 75 M. prevalent cases, 27 M. incident cases and 17 M. cancer-related deaths by 2030 (Adeloye et al., 2016). The cancer prevalence is gender-dependent with men presenting higher incidence rates for tumors of the lung (16.7%), prostate (15.0%), colorectum (10.0%), stomach (8.5%) and liver (7.5%), while women reveal more cases of breast (25.2%), colorectum (9.2%), lung (8.7%), cervix (7.9%), and stomach (4.8%). North Africa and Middle-East acquaint worldwide for 3.8% of the new cancer cases and for 4.1% for cancer-related deaths. According to WHO, the raw incidence of cancer in Morocco was of 123.1 per 100,000 habitants in men and of 77.5 per 100,000 in women (Observatory)^1^ in 2012. Throughout all North African countries, the prevalence in men are higher than in women, with Egypt as country with the highest prevalence (145.9 for men and 100.5 for women) and Tunisia with the lowest prevalence (96.6 for men and 52.9 for women).

Since immemorial times, human beings acquired knowledge on the medicinal use of plants (El-Seedi et al., 2013; Ouelbani et al., 2016). Those plants have been extensively applied in folk medicine to treat ailments and diseases (El-Seedi et al., 2013) and are still used in the rural areas of developing countries (El-Seedi et al., 2013; Ouelbani et al., 2016). In fact, World Health Organization (WHO) reported that around 80% of the world population still relies on plants as source for primary health care (Cordell, 1995), while traditional medicine is the only health source available for 60% of the global population (El-Seedi et al., 2013). Medicinal plants are frequently the only form of cancer treatment for many people in North Africa, either due to low income or spatial distance from the urban treatment centers (Kabbaj et al., 2012).

The aim of this review is to compile data about the anticancer potential of plants found in ethnobotanical surveys of North African countries. The information was collected in several scientific research engines, *PubMed, Science Direct, Scopus, Web of Science*, and *Google Scholar* comprising studies conducted between 2000 and 2016.

In addition to ethnobotanical surveys, this review also includes experimental evidence on the cytotoxic effects of medicinal plants as well as their cellular and molecular mechanisms in cancer cells.

## Ethnobotanical studies

**Figure 1:**
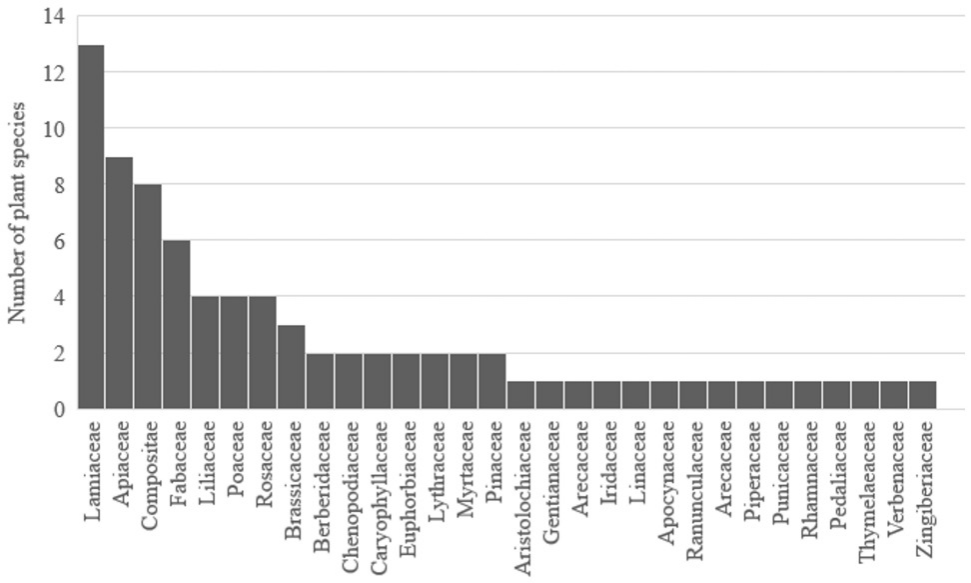
Plant families and species traditionally used for cancer therapy in North African folk medicines.

The ethnobotanical surveys were predominantly found in Morocco and Algeria. In other North African countries (Egypt, Tunisia, and Libya), less is known about the cytotoxic properties of medicinal plants against cancer cells. Table 1 compiles the botanical information, the geographical location, the type of therapy, the cancer types investigated, the plants' parts used (e.g., leaf, aerial parts, seeds), and the preparation method (e.g., infusion, decoction). In those cases, where information was obtained from either the general population or herbalists/botanists we considered cancer therapy as monotherapy (i.e., only medicinal plants). If information was gathered from patients in cancer treatment centers the therapy was considered to be a combination of complementary and standard chemo/radiotherapy (co-therapy). The most predominant botanical families used as anticancer agents were Lamiaceae (13 species), Apiaceae (9 species), Compositae (8 species), and Fabaceae (6 species; Figure 1). Two surveys conducted at the National Institute of Oncology in Rabat (Morocco) showed that the most used plants by the patients were *Nigella sativa* L. (Ranunculaceae), *Trigonella foenum-graecum* L. (Fabaceae), *Aristolochia longa* L. (Aristolochiaceae), *Marrubium vulgare* L. (Lamiaceae), and *Cassia absus* L. (Fabaceae) (Kabbaj et al., 2012; Chebat et al., 2014). Recently, a review was conducted on the anticancer potential of plants used in the Arabian and Islamic world (Ahmad et al., 2016) which included *N. sativa*. However, this work did not mention the countries in which they are used.

**Table 1:** Plants used by herbalists for cancer therapy in North Africa.

**Plant**	**Family**	**Origin**	**Part used**	**Type Prep**.	**Therapy type**	**Cancer**	**References**
*Agave americana*	Agavaceae	Morocco			Co-therapy	Lung	Chebat et al., 2014
*Ajuga iva*	Lamiaceae	Algeria					Benarba et al., 2015; Ouelbani et al., 2016
		Morocco	Rod, Leaf	Grind w/Honey	Co-therapy	Breast	Kabbaj et al., 2012
					Co-therapy	Stomach	Chebat et al., 2014
*Allium cepa*	Liliaceae	Morocco	Bulb	Brut	Co-therapy	All types	Kabbaj et al., 2012
*Allium sativum*	Liliaceae	Morocco	Bulb	Brut	Co-therapy	All types	Kabbaj et al., 2012
					Co-therapy	Blood, Lung and Stomach	Chebat et al., 2014
*Aloe ferox*	Liliaceae	Morocco	Leaf	Infusion	Co-therapy	Digestive	Kabbaj et al., 2012
*Aloe socotrina*	Liliaceae	Algeria					Benarba et al., 2015
		Morocco	Cortex, Leaf	Decoction, Oil	Monotherapy		Jamila and Mostafa, 2014
					Co-therapy	Neck	Chebat et al., 2014
*Ammodaucus leucotrichus*	Apiaceae	Morocco	Seed	Grind w/Honey	Co-therapy	Lung	Kabbaj et al., 2012
*Anacyclus pyrethrum*	Asteraceae	Algeria					Benarba et al., 2015; Ouelbani et al., 2016
*Anastatica hierochuntica*	Brassicaceae	Morocco			Co-therapy	Neck and esophagus	Chebat et al., 2014
*Anchusa azurea*	Borraginaceae	Algeria	Aerial parts	Infusion	Monotherapy		Boudjelal et al., 2013
*Anethum graveolens*	Apiaceae	Algeria	Aerial parts	Infusion	Monotherapy		Boudjelal et al., 2013
*Apium graveolens*	Apiaceae	Morocco	Leaf	Decoction	Co-therapy	Kidney, Digestive	Kabbaj et al., 2012
*Argania spinosa*	Sapotaceae	Morocco	Seed	Extraction	Co-therapy	Skin	Kabbaj et al., 2012
*Argania spinosa*	Sapotaceae	Morocco			Co-therapy	Breast and Ovary	Chebat et al., 2014
*Aristolochia longa*	Aristolochiaceae	Morocco	Rhizome	Powder	Monotherapy		Jamila and Mostafa, 2014
			Root	Grind w/Honey	Co-therapy	All types	Kabbaj et al., 2012
					Co-therapy	Breast, Brain, Lung, Skin, Colon, Blood, Ovary and Stomach	Chebat et al., 2014
*Artemisia absinthium*	Asteraceae	Morocco	Leaf	Infusion	Co-therapy	Digestive	Kabbaj et al., 2012
*Artemisia herba-alba*	Asteraceae	Algeria					Ouelbani et al., 2016
			Aerial parts	Infusion	Co-therapy	Digestive	Kabbaj et al., 2012
					Co-therapy	Stomach and Kidney	Chebat et al., 2014
*Artemisia vulgaris*	Asteraceae	Morocco	Aerial parts	Infusion	Co-therapy	Digestive	Kabbaj et al., 2012
*Atriplex halimus*	Amaranthaceae	Algeria					Benarba et al., 2015
*Berberis hispanica*	Berberidaceae	Morocco	Cortex, Whole Plant, Roots	Powder, Infusion, Decoction	Monotherapy		Jamila and Mostafa, 2014
					Co-therapy	Blood, Pancreas and Breast	Chebat et al., 2014
*Berberis vulgaris*	Berberidaceae	Algeria					Benarba et al., 2015
*Borago officinalis*	Boraginaceae	Morocco	Stamen	Grind w/Honey	Co-therapy	All types	Kabbaj et al., 2012
*Brassica* spp.	Brassicaceae	Morocco			Co-therapy	Breast	Chebat et al., 2014
*Capparis spinosa*	Capparaceae	Morocco	Fruit	Grind w/Honey	Co-therapy	Lymphoma	Kabbaj et al., 2012
					Co-therapy	Bone and Breast	Chebat et al., 2014
*Caralluma europea*	Asclepiadaceae	Morocco			Co-therapy	Breast	Chebat et al., 2014
*Carthamus tinctorius*	Asteraceae	Morocco			Co-therapy	Breast	Chebat et al., 2014
*Carum carvi*	Apiaceae	Morocco	Seed	Grind w/Honey	Co-therapy	Lung	Kabbaj et al., 2012
					Co-therapy	Stomach	Chebat et al., 2014
*Cassia absus*	Fabaceae	Morocco	Seed	Grind w/Honey	Co-therapy	All types	Kabbaj et al., 2012
*Centaurium erythreae*	Gentianaceae	Algeria					Ouelbani et al., 2016
*Ceratonia siliqua*	Fabaceae	Morocco			Co-therapy	Lung	Chebat et al., 2014
		Algeria					Ouelbani et al., 2016
*Chamaerops humilis*	Arecaceae	Morocco			Co-therapy	Brain and Blood	Chebat et al., 2014
*Chenopodium ambrosioides*	Chenopodiaceae	Morocco	Leaf	Decoction	Co-therapy	Amygdale	Kabbaj et al., 2012
*Cicer arietinum*	Fabaceae	Morocco	Seed	Grind w/ Honey	Co-therapy	Lung	Kabbaj et al., 2012
					Co-therapy	Lung and Liver	Chebat et al., 2014
*Cichorium intybus*	Asteraceae	Algeria					Benarba et al., 2015
*Citrullus colocynthis*	Cucurbitaceae	Algeria	Aerial parts, Fruits	Decoction, pomade	Monotherapy		Boudjelal et al., 2013
*Coix lacryma-joib*	Poaceae	Morocco			Co-therapy	Breast and Kidney	Chebat et al., 2014
*Coriandrum sativum*	Apiaceae	Morocco	Aerial parts	Grind w/Honey	Co-therapy	Kidney, Digestive	Kabbaj et al., 2012
*Corrigiola telephiifolia*	Caryophyllaceae	Morocco	Root	Decoction	Co-therapy	Liver, Digestive	Kabbaj et al., 2012
*Crataegus azarolus*	Rosaceae	Algeria					Ouelbani et al., 2016
*Crocus sativus*	Iridaceae	Algeria					Ouelbani et al., 2016
		Morocco			Co-therapy	Blood	Chebat et al., 2014
			Stamen	Decoction	Co-therapy	All types	Kabbaj et al., 2012
*Cuminum cyminum*	Apiaceae	Morocco	Seed	Grind w/ Honey	Co-therapy	Lung	Kabbaj et al., 2012
					Co-therapy	Neck	Chebat et al., 2014
*Daucus carota*	Apiaceae	Morocco	Root	Decoction	Co-therapy	Kidney, Digestive	Kabbaj et al., 2012
*Dittrichia viscosa*	Asteraceae	Morocco			Co-therapy	Stomach	Chebat et al., 2014
*Equisetum arvense*	Equisetaceae	Algeria	Aerial parts	Decoction	Monotherapy		Boudjelal et al., 2013
*Euphorbia beaumeriana*	Euphorbiaceae	Morocco			Co-therapy	Skin	Chebat et al., 2014
*Euphorbia resinifera*	Euphorbiaceae	Morocco	Aerial parts	Grind w/Honey	Co-therapy	All types	Kabbaj et al., 2012
*Ficus carica*	Moraceae	Morocco	Fruit	Brut	Co-therapy	Digestive	Kabbaj et al., 2012
*Foeniculum vulgare*	Apiaceae	Morocco	Seed	Decoction	Co-therapy	Digestive	Kabbaj et al., 2012
					Co-therapy	Stomach	Chebat et al., 2014
*Globularia alypum*	Globulariaceae	Morocco			Co-therapy	Stomach	Chebat et al., 2014
*Glycyrriza glabra*	Fabaceae	Morocco			Co-therapy	Blood and Lung	Chebat et al., 2014
*Haloxylonsco parium*	Chenopodiaceae	Morocco	Leaf, Fruit	Decoction	Co-therapy	Liver	Kabbaj et al., 2012
*Herniaria glabra*	Caryophyllaceae	Morocco	Aerial parts	Decoction	Co-therapy	Renal, Digestive	Kabbaj et al., 2012
*Insula viscosa*	Asteraceae	Morocco	Leaf/Root	Decoction	Monotherapy		El-Hilaly et al., 2003
			Leaf, Flower	Grind w/ Honey	Co-therapy	Breast	Kabbaj et al., 2012
*Laurus nobilis*	Lauraceae	Algeria					Ouelbani et al., 2016
*Lavandula officinalis*	Lamiaceae	Morocco	Leaf	Infusion	Co-therapy	Urogenital system	Kabbaj et al., 2012
					Co-therapy	Neck and Stomach	Chebat et al., 2014
*Lawsonia inermis*	Lythraceae	Algeria					Benarba et al., 2015
		Morocco	Flower	Cataplasm	Co-therapy	Skin	Kabbaj et al., 2012
					Co-therapy	Breast and Ovary	Chebat et al., 2014
*Lepidium sativum*	Brassicaceae	Morocco	Seed	Grind w/Honey	Co-therapy	Lung, Digestive	Kabbaj et al., 2012
					Co-therapy	Ovary	Chebat et al., 2014
*Linum usitatissimum*	Linaceae	Algeria					Ouelbani et al., 2016
		Morocco	Seed	Grind w/Honey	Co-therapy	Lymphoma	Kabbaj et al., 2012
					Co-therapy	Blood, Liver and Stomach	Chebat et al., 2014
*Marrubium vulgare*	Lamiaceae	Morocco	Rod, Leaf	Decoction	Co-therapy	Digestive	Kabbaj et al., 2012
					Co-therapy	Breast, Stomach and Ovary	Chebat et al., 2014
			Leaf, Stem	Infusion, Ingestion, pomade, chewing, washing	Monotherapy		Teixidor-Toneu et al., 2016
*Mentha pulegium*	Lamiaceae	Morocco	Rod, Leaf	Infusion	Co-therapy	Gingival	Kabbaj et al., 2012
					Co-therapy	Kidney	Chebat et al., 2014
*Mentha suaveolens*	Lamiaceae	Morocco			Co-therapy	Abdominal tumefaction	Chebat et al., 2014
*Myrtus communis*	Myrtaceae	Algeria					Benarba et al., 2015; Ouelbani et al., 2016
		Morocco	Leaf	Decoction	Co-therapy	Digestive	Kabbaj et al., 2012
*Nerium oleander*	Apocynaceae	Algeria					Ouelbani et al., 2016
		Morocco	Leaf	Mouthwash solution	Co-therapy	Gingival	Kabbaj et al., 2012
					Co-therapy	Neck and Armpit	Chebat et al., 2014
*Nigella sativa*	Ranunculaceae	Morocco	Seed	Grind w/Honey	Co-therapy	All types	Kabbaj et al., 2012
					Co-therapy	Breast, Liver, Kidney, Stomach, Lung and Brain	Chebat et al., 2014
			Seed	Ingestion, Inhalation	Monotherapy		Teixidor-Toneu et al., 2016
*Olea europaea*	Oleaceae	Morocco	Fruit	Extraction	Co-therapy	Lung	Kabbaj et al., 2012
*Origanum compactum*	Lamiaceae	Morocco	Rod, Leaf	Infusion	Co-therapy	Digestive, Gingival	Kabbaj et al., 2012
					Co-therapy	Neck, Lung and Kidney	Chebat et al., 2014
*Origanum majorana*	Lamiaceae	Morocco/Algeria	Leaf	Infusion, Decoction	Monotherapy		Jamila and Mostafa, 2014; Ouelbani et al., 2016
*Panax ginseng*	Araliaceae	Morocco	Leaf	Grind w/Honey	Co-therapy	Lung	Kabbaj et al., 2012
*Peganum harmala*	Zygophyllaceae	Morocco	Seed	Grind w/Honey	Co-therapy	All types	Kabbaj et al., 2012
					Co-therapy	Breast, Liver and Bones	Chebat et al., 2014
*Pennisetum typhoides*	Poaceae	Morocco			Co-therapy	Neck	Chebat et al., 2014
*Petroselinum crispum*	Apiaceae	Morocco	Aerial parts	Decoction	Co-therapy	Kidney	Kabbaj et al., 2012
*Phoenix dactylifera*	Arecaceae	Morocco	Fruit	Brut	Co-therapy	Lymphoma	Kabbaj et al., 2012
					Co-therapy	Lung and Brain	Chebat et al., 2014
*Pimpinella anisum*	Apiaceae	Morocco	Seed	Decoction	Co-therapy	Kidney, Digestive	Kabbaj et al., 2012
*Pinus halepinsis*	Pinaceae	Morocco	Seed	Extraction	Co-therapy	Esophagus	Kabbaj et al., 2012
*Pinus sylvestris*	Pinaceae	Algeria					Ouelbani et al., 2016
*Piper nigrum*	Piperaceae	Algeria					Benarba et al., 2015
		Morocco			Co-therapy	Pancreas	Chebat et al., 2014
*Pistacia lentiscus*	Anacardiaceae	Morocco	Leaf	Brut, Decoction	Co-therapy	Digestive	Kabbaj et al., 2012
*Prunus armenica*	Rosaceae	Morocco	Leaf, Flower, Seeds	Decoction, Powder, Oil	Monotherapy		Jamila and Mostafa, 2014
*Prunus persica*	Rosaceae	Algeria					Benarba et al., 2015
*Punica granatum*	Lythraceae	Morocco	Rind	Decoction	Co-therapy	Skin	Kabbaj et al., 2012
					Co-therapy	Liver, Lung and Breast	Chebat et al., 2014
*Rhamnus alaternus* subsp. *alaternus*	Rhamnaceae	Algeria					Ouelbani et al., 2016
*Rheum palmatum*	Polygonaceae	Algeria	Aerial Parts	Infusion, Decoction	Monotherapy		Ouelbani et al., 2016
*Rosa canina*	Rosaceae	Morocco			Co-therapy	Breast	Chebat et al., 2014
*Rosmarinus officinalis*	Lamiaceae	Algeria					Ouelbani et al., 2016
		Morocco	Leaf	Decoction	Co-therapy	Digestive	Kabbaj et al., 2012
					Co-therapy	Stomach and Lung	Chebat et al., 2014
*Salvia officinalis*	Lamiaceae	Morocco	Leaf	Infusion	Co-therapy	Intestine, Lungs	Kabbaj et al., 2012
*Sesamum indicum*	Pedaliaceae	Morocco			Co-therapy	Breast	Chebat et al., 2014
		Algeria					Benarba et al., 2015
*Sorghum* spp.	Poaceae	Morocco			Co-therapy	Lung	Chebat et al., 2014
*Syzygium aromaticum*	Myrtaceae	Morocco			Co-therapy	Bowel	Chebat et al., 2014
*Tetraclinis articulata*	Cupressaceae	Morocco			Co-therapy	Liver	Chebat et al., 2014
*Thymelaea lathryroides*	Thymelaeaceae	Morocco	Aerial parts	Decoction	Co-therapy	Uterus	Kabbaj et al., 2012
*Thymus* spp.	Lamiaceae	Morocco	Rod, Leaf	Infusion	Co-therapy	Digestive	Kabbaj et al., 2012
*Trigonella foenum-graecum*	Fabaceae	Morocco	Seed	Grind w/ Honey	Co-therapy	Digestive	Kabbaj et al., 2012
					Co-therapy	Brain, Lung and Stomach	Chebat et al., 2014
*Triticum repens*	Poaceae	Algeria					Benarba et al., 2015
*Urginea maritima*	Asparagaceae	Algeria					Ouelbani et al., 2016
		Morocco	Leaf, Root	Decoction	Monotherapy		Merzouki et al., 2000
*Urtica dioica*	Urticaceae	Algeria					Ouelbani et al., 2016
*Verbena officinalis*	Verbenaceae	Morocco	Leaf	Infusion	Co-therapy	Gallbladder	Kabbaj et al., 2012
*Vicia faba*	Fabaceae	Morocco	Seed	Grind w/Honey	Co-therapy	Lung	Kabbaj et al., 2012
*Vitis vinifera*	Vitaceae	Algeria					Benarba et al., 2015; Ouelbani et al., 2016
*Zingiber officinale*	Zingiberiaceae	Morocco	Root	Grind w/Honey	Co-therapy	All types	Kabbaj et al., 2012
					Co-therapy	Breast	Chebat et al., 2014

**Figure 2:**
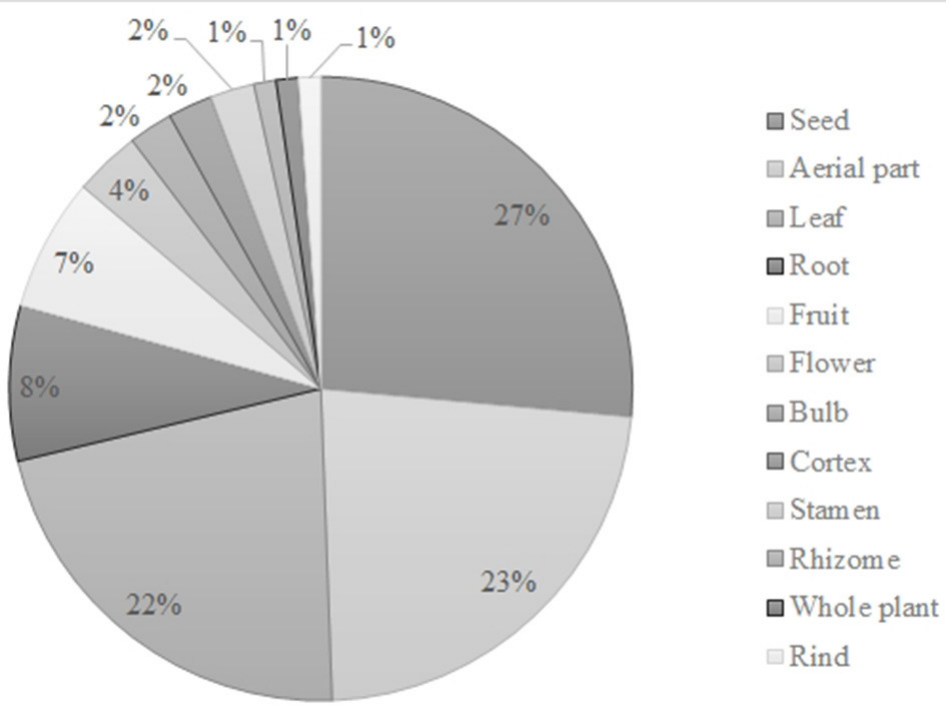
Plant parts used for cancer therapy in North African traditional medicines.

**Figure 3:**
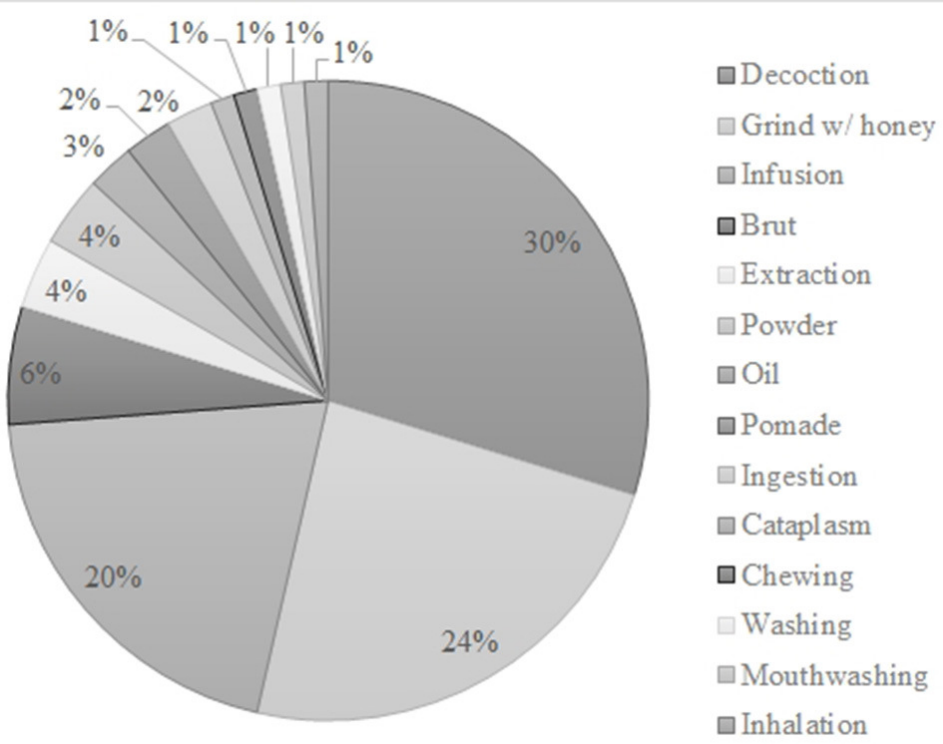
Preparation methods ascribed in several ethnobotanical surveys in North Africa.

The plant parts used as well as the preparation methods of anticancer agents were as diverse as the plants themselves. The most common plant parts were the seeds (23) followed by the aerial parts (20) and leaves (19) (Figure 2). Regarding the preparation methods, the most described are decoction (25), grinded with honey (20) and infusion (17) (Figure 3).

## Scientific evidence and mechanisms of action

Despite the widespread use of medicinal plants in North Africa, many species still lack scientific prove of their anticancer activity. In fact, Kabbaj et al. (2012) described that 55 plants are used by patients at the National Institute of Oncology at Rabat (Morocco) albeit only 28 have been previously described for their cytotoxic properties against tumor cells. This chapter focusses, first and foremost, on (1) *in vitro* cytotoxicity against cancer cell lines for both volatile and non-volatile extracts and isolated major phytochemicals and (2) *in vivo* assays in those cases, where such studies were carried out. Afterwards, potential mechanisms of action for both extracts and isolated compounds will be reviewed, mainly cell cycle arrest, cell death induction and signal transduction pathways as well as invasiveness and migration of cancer cells.

### *In vitro* cytotoxicity assays

Of all used plants in North African folk medicine, *N. sativa* is one of the scientifically best analyzed. A plethora of cancer cell lines have been used for the determination of cytotoxicity of medicinal plants. Of those, breast cancer cell lines (MCF-7, MCF-7/Dox and MCF-7/Topo; MDA-MB-231), colon carcinoma (HCT 116), hepatocellular carcinoma (HepG2), cervix carcinoma (Hep-2), prostate cancer (PC-3), and lung carcinoma (A549) are the most commonly used ones.

#### *Nigella sativa* and thymoquinone

*N. sativa* (Ranunculaceae) is known as “Habbat Al-barakah” in Arabic and black cumin or black seed in English. This plant is widely used in Arabic medicine to treat several ailment including, but not limited to, cancer (Randhawa and Alghamdi, 2011). Seed oil from non-heated seeds of *N. sativa* decreased the growth rate of MC38 (mouse colon carcinoma) cell line by 40% (IC_50_ = 1.4 μg/mL), while oil from seeds heated at 50°C decreased the growth rate by 90% (IC_50_ = 0.6 μg/mL; Agbaria et al., 2015). In another study, the essential oil, an ethyl acetate and a butanol extracts were tested against several cancer cell lines. The essential oil which mainly consisted of thymoquinone (62.17%), carvacrol (8.29%) and 2-methyl-5-prop-enyldihydroquinone and an ethyl acetate extract rich in monoxideterpenes was strongly cytotoxic against P815 (IC_50_ = 0.6 and 0.75%, respectively), Vero (IC_50_ = 0.2 and 0.22%, respectively), BSR (IC_50_ = 1.2 and 0.2%, respectively) and ICO1 cells. The butanol extract, characterized by two saponosides derived from α-amyrin, exerted weak activity against these cell lines (IC_50_ ~2%) except for the ICO1 cell line (IC_50_ = 0.26%; Ait Mbarek et al., 2007). Several supercritical carbon dioxide extracts from the seeds of *N. sativa* were tested against HCT 116 (61.11–88.80% cell viability), MCF-7 (0.07–83.95% cell viability), MDA-MB-231 (85.58 and 88.22% cell viability), HepG2 (91.59% cell viability), PC-3 (86.05–90.08% cell viability) and CCD-18Co cells (69.70–88.70% cell viability; Baharetha et al., 2013). The most potent extract (60°C and 2500 psi) showed a dose-dependent activity in MCF-7 cells with an IC_50_ value of 53.34 ± 2.15 μg/mL. An aqueous extract from the seeds decreased HepG2 cell survival (IC_50_ = 7 mg/mL) and cell activity (IC_50_ = 6 mg/mL; Thabrew et al., 2005). The ethyl acetate fraction obtained from an ethanolic extract from the seeds was cytotoxic toward Molt4 (IC_50_ = 12 μg/mL) and P388 lymphocytic leukemia cells (IC_50_ = 17 μg/mL), J82 bladder carcinoma cells (IC_50_ = 22 μg/mL), Wehi 184 fibrosarcoma cells (IC_50_ = 14 μg/mL), LL/2 Lewis lung carcinoma cells (IC_50_ = 16 μg/mL), SW620 lymph node metastasis of colon adenocarcinoma (IC_50_ = 18 μg/mL), and HepG2 hepatocellular carcinoma cells (IC_50_ = 11 μg/mL; Swamy and Tan, 2000). The chloroform-methanol eluate obtained from an ethyl acetate fraction of an ethanolic extract demonstrated a selective inhibition toward HepG2 (IC_50_ = 8 μg/mL), Molt4 (IC_50_ = 10 μg/mL) and LL/2 cells (IC_50_ = 11 μg/mL). Two extracts, aqueous and petroleum ether, were effective against both HepG2 (IC_50_ = 300 and 710 μg/mL, respectively) and MCF-7 (IC_50_ = 180 and 435 μg/mL, respectively), while the chloroform extract only inhibited MCF-7 (IC_50_ = 522 μg/mL; Sadiq et al., 2015). The essential oil from plants of Tunisia with high amounts of *p*-cymene demonstrated a dose-dependent effect against Hep-2 cell line with an IC_50_ = 55.2 μg/mL (Jrah Harzallah et al., 2011). Another oil from Tunisian *N. sativa* was effective against A549 lung carcinoma and DLD-1 colon adenocarcinoma cells showing IC_50_ values of 43 and 46 μg/mL, respectively (Bourgou et al., 2010). Islam et al. (2004) tested the anticancer efficacy of the essential oil against four stomach cancer cell lines. Of all tested lines, SCL-37'6 was the most sensitive one (IC_50_ = 120.40 μg/mL). A different volatile extract from the seeds obtained with petroleum ether decreased the viability of A549 lung carcinoma cells exposed to concentrations above 0.1 mg/mL, while an alcoholic extract only exhibited toxicity at concentrations equal or higher than 0.25 mg/mL (Al-Sheddi et al., 2014). A nanoemulsion of essential oil was cytotoxic to MCF-7 breast cancer cells in a dose- and time-dependent manner (IC_50_ = 82 and 59 μL/mL for 24 and 48 h, respectively; Periasamy et al., 2016).

**Figure 4:**
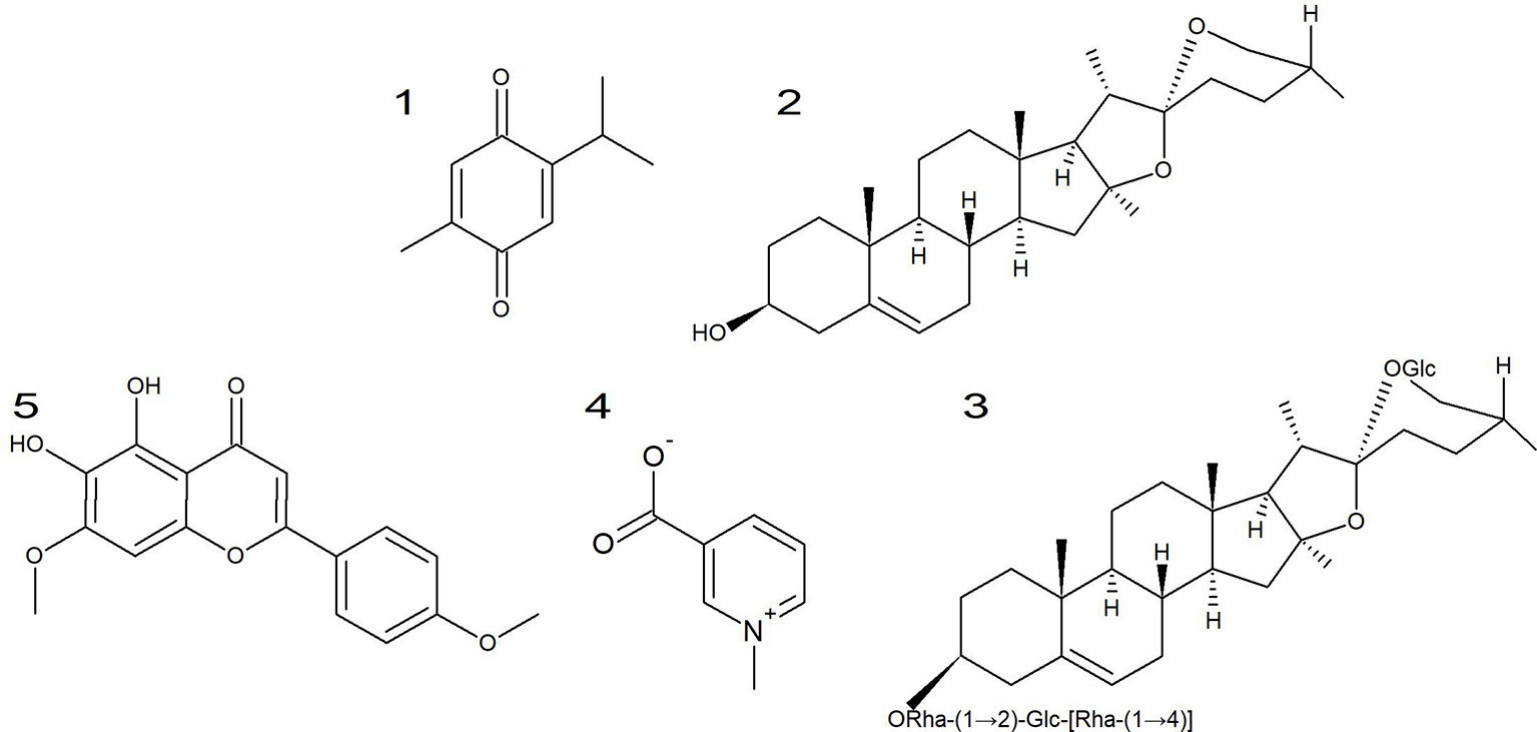
Chemical structures of the major compounds from selected North African plants. (1) Thymoquinone from *N. sativa*. (2) diosgenin, (3) protodioscin, and (4) trigonelline from *T. foenum-graecum*, (5) ladanein from *M. vulgare*.

The bioactive properties of *N. sativa* are usually associated with the content in thymoquinone (Figure 4, 1) (Agbaria et al., 2015) that have been widely described as anti-inflammatory, antioxidant and anti-neoplasic (Paramasivam et al., 2012; Raghunandhakumar et al., 2013; Agbaria et al., 2015). In fact, the strongest anticancer activity was achieved in seed oils heated between 50 and 150°C with the highest content of thymoquinone (Agbaria et al., 2015). The compound significantly inhibited the growth of Hep-2 cells in a dose-dependent manner with an IC_50_ of 19.25 μg/mL (Jrah Harzallah et al., 2011). It was also cytotoxic toward A549 lung carcinoma and DLD-1 colon adenocarcinoma cells (IC_50_ = 13 and 5.9 μM, respectively; Bourgou et al., 2010). Khalife et al. (2016) described that the cytotoxic effect was dose- and time-dependent (IC_50_ = 59.2 and 68.4 μM, for 24 and 48 h, respectively) for HT-29 colorectal carcinoma cells. Woo et al. (2011) described the cytotoxic effect of thymoquinone against different breast cancer lines with MDA-MB-231 cells as the most susceptible ones (IC_50_ = 11 μM after 48 h). Several breast cancer cell lines were described as susceptible to thymoquinone in a dose- and time-dependent manner, being T-47D and MDA-MB-468 the most susceptible (IC_50_ = 18.06 and 12.30 μM after 48 h, respectively; Rajput et al., 2013). Arafa et al. (2011) demonstrated that this compound inhibited cell proliferation in a doxorubicin-resistant breast cancer cell line, MCF-7/DOX (65% inhibition after 48 h with 100 μM). It also inhibited the growth of squamous cell carcinoma cells (A431, Hep2, and RPMI 2650; Das et al., 2012). In addition, it also dose- and time-dependently inhibited the growth of HCT116 colon cancer cells (Kundu et al., 2014). Alhosin et al. (2010) demonstrated that thymoquinone successfully inhibited proliferation (IC_50_ = 24.2 μM after 24 h vs. 23.3 μM after 48 h) and viability (IC_50_ = 24.3 μM after 24 h vs. 23.1 μM after 48 h) in p53-defected Jurkat lymphoblastic leukemia cells in a dose- and time-dependent manner. Thymoquinone was also cytotoxic to neuroblastoma Neuro-2a cells in a dose-and time-dependent manner (IC_50_ = 40 and 36 μM after 24 and 48 h treatment; Paramasivam et al., 2012). Racoma et al. (2013) described that thymoquinone was cytotoxic to several glioblastoma cell lines with Gli36ΔEGFR as the most susceptible one (IC_50_ = 2.4 μM). In another study, it was able to induce cell death in both a DNA-PKcs-wild-type (M059K) and -mutant (M059J) glioblastoma cell lines being the former more susceptible than the latter (Gurung et al., 2010). Zubair et al. (2013) demonstrated the inhibitory effect on the proliferation of different prostate cancer lines in a dose-dependent manner. Effenberger et al. (2010) investigated the anticancer activity of thymoquinone and its terpene-conjugated derivatives against several cancer cell lines. Thymoquinone demonstrated a good cytotoxicity toward 518A2 (IC_50_ = 28.3 μM), HL-60 (IC_50_ = 27.8 μM), KB-V1/Vbl (IC_50_ = 32.3 μM) and MCF-7/Topo cells (IC_50_ = 26.7 μM). Conjugation with (-)-menthol greatly improved the effectiveness against cancer cell lines with IC_50_ values of 3.9, 9.0, 7.0, and 5.4 μM against 518A2, HL-60, KB-V1/Vbl, and MCF-7/Topo, respectively. The addition of C_6_-spacer between the quinone and the terpene moiety decreased the anticancer activity of the compound if compared with a shorter spacer (e.g., IC_50_ = 11.7 vs. 9.0 μM against HL-60) although it was stronger than thymoquinone (IC_50_ = 11.7 vs. 27.8 μM against HL-60). The addition of a C_9_ or higher spacers completely nullified the activity (IC_50_ > 100 vs. 27.8 μM for HL-60). By contrast, the conjugation with a betulinic acid moiety decreases the activity against all tested cell lines except HL-60 (IC_50_ = 13.7 vs. 27.8 μM). In contrast to the remaining compounds, the addition of a C_6_-spacer between the betulinic moiety and the quinone improved the cytotoxic activity of the derivative (IC_50_ = 0.13 vs. 13.7 μM for HL-60). Thymoquinone demonstrated weak activity against MCF-7 cells (IC_50_ = 109.15 μg/mL after 72 h treatment; Dehghani et al., 2015). The nanoemulsification of this compound considerably improved the cytotoxic effect (IC_50_ = 46.78 μg/mL after 72 h treatment).

#### *Trigonella foenum-graecum*, diosgenin, and protodioscin

*T. foenum-graecum* (Fabaceae) is known as “helba” in Arabic (Hammiche and Maiza, 2006) and fenugreek in English (Abdel-Barry et al., 1997). This plant have been described as possessing several pharmacological activities, including anticancer (Yadav and Baquer, 2014). In fact, a recent paper reviews the anticancer potential of both *T. foenum-graecum* extracts and isolated compounds (El Bairi et al., 2017) describing the effects on the major hallmarks of cancer, sustaining proliferative signaling, angiogenesis, cell death evasion, tumor promoting inflammation, invasion, and metastasis and genomic instability. A water extract obtained from the seeds was cytotoxic toward several cancer cell lines (T-cell lymphoma, B-cell lymphoma, thyroid papillary carcinoma, and breast cancer; Alsemari et al., 2014). A crude methanol extract from the seeds induced cell death in a dose-dependent manner (IC_50_ = 1,000 μg/mL; Khalil et al., 2015), however, significant inhibition was achieved only at concentrations of 100 μg/mL. Seed extracts were cytotoxic to a plethora of breast, pancreatic and prostate cancer cell lines (Shabbeer et al., 2009). Crude extracts of fenugreek decreased the cell viability toward both drug-sensitive and drug-resistant cancer cell lines (Saeed et al., 2015). Methanol extracts from the whole plant induced cytotoxicity in MCF-7 breast cancer cells (IC_50_ = 65 μg/mL; Alshatwi et al., 2013). In the same cell model, a chloroform extract also induced cytotoxicity (IC_50_ = 41.6 μg/mL; Khoja et al., 2011). Both aqueous and ethanolic extracts exerted cytotoxic effects against MCF-7 cells in a dose- and time-dependent manner (Sebastian and Thampan, 2007), while an ethanolic extract induced cell death in Jurkat leukemia cells (Al-Daghri et al., 2012). Seed oil from fenugreek induced cytotoxicity toward several cancer cell lines [HEp2 (human epidermoid cancer cells), WISH, and MCF-7; Al-Oqail et al., 2013].

Diosgenin (Figure 4, 2), the major compound of fenugreek, reduced cell proliferation in a time- and dose-dependent manner in HT-29 colon cancer cells (Raju et al., 2004). Furthermore, it induced cell death to several breast, pancreatic and prostate cancer cell lines (Shabbeer et al., 2009). In PC-3 prostate cancer cells, it inhibited the growth at 50 μM in a time-dependent manner (Chen et al., 2011). Diosgenin (25 μM) induced cytotoxicity and inhibited cell proliferation in KBM-5 cells (Shishodia and Aggarwal, 2006). Furthermore, co-treatment with chemotherapeutic agents potentiated the cytotoxic effect of the latter. The saponin revealed inhibitory effects on several squamous cell carcinomas (Das et al., 2012). Diosgenin inhibited the growth of A549 lung cancer cells in a dose- and time-dependent manner (47 and 43 μM after 72 h, respectively; Rahmati-Yamchi et al., 2014). Treatment with diosgenin induced cell death in HEL erythroleukemia cells (90% inhibition at 40 μM after 48 h; Leger et al., 2004). In a different leukemia model, K562, it also induced cytotoxicity (IC_50_ = 15 μM; Liu et al., 2005). The 1547 osteosarcoma cell line is susceptible to this compound (86% inhibition at 40 μM for 24 h; Moalic et al., 2001).

Protodioscin (Figure 4, 3), isolated from fenugreek, inhibited the growth of HL-60 leukemic cells (100% inhibition at 10 μM), while demonstrating poor inhibitory effects against KATO III gastric cancer cells (42.5% at 10 μM; Hibasami et al., 2003). In addition, this compound was cytotoxic toward 60 cell lines from the National Cancer Institute (IC_50_ = 1.64 - >100 μM; Hu and Yao, 2002). Furthermore, it was cytotoxic in a dose-dependent manner toward HCT116 (IC_50_ = 2.26 μM), HT-29 (IC_50_ = 3.48 μM), SW480 and EMT6 (IC_50_ = 6.68 μM) and DU145 cells (IC_50_ = > 28.63 μM; Manase et al., 2012).

#### Aristolochia longa

*A. longa* (Aristolochiaceae) is known as “Berrostom” in Algeria. This plant is widely used in traditional medicine including cancer treatment (Benarba et al., 2016). Nevertheless, very few studies have been conducted on the anticancer activity of this plant. An aqueous extract of *A. longa* reduced cell viability of BL41 Burkitt's lymphoma cells (IC_50_ = 15.63 μg/mL; Benarba et al., 2012) as well as of two triple-negative breast cancer cell lines, MDA-MB-231 (IC_50_ = 97 μg/mL) and HBL100 (IC_50_ = 40 μg/mL; Benarba et al., 2016).

#### *Marrubium vulgare* and ladanein

*M. vulgare* (Lamiaceae) is known as “merriwa” (Tahraoui et al., 2007), “ifzi” (Teixidor-Toneu et al., 2016), “amarriw” or “ifza” (Merzouki et al., 2000), “marrîwet” (Kabbaj et al., 2012) in several Arab-speaking countries or as “horehound” in English (Tahraoui et al., 2007). This plant is widely used as treatment for several ailments including cancer (Paunovic et al., 2016). Indeed, the essential oil from *M. vulgare* decreased the cell viability in a dose-dependent manner (IC_50_ = 0.258 μg/mL; Zarai et al., 2011). An ethanolic extract obtained from *M. vulgare* exerted cytotoxicity in a dose-dependent manner in B16 melanoma and U251 glioma cells (Paunovic et al., 2016).

**Figure 5:**
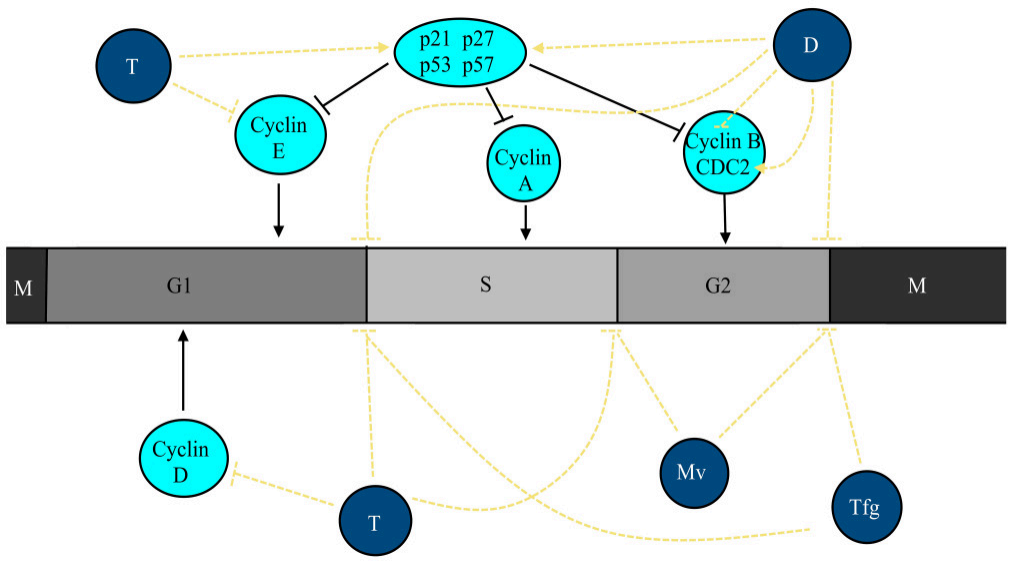
Extracts from plants used for cancer therapy in North African countries and major compounds are able to cause cell cycle arrest in cancer cells. Thymoquinone (T) causes cell cycle arrest at G1 and S phases and decreases the activity/expression of cyclin D1 and E while promoting the activity of p53 and p27. Extracts from *T. foenum-graecum* (Tgf) and diosgenin (D) causes the cell cycle to stop at G1 and G2/M phases. In addition, diosgenin (D) blocks the activity of cyclin B1 while increasing the activity/expression of p21, p53, and CDC2. Extracts from *M. vulgare* (Mv) blocks cell cycle progression at S and G2/M phases.

Ladanein (Figure 4, 5), a compound found in *M. vulgare* have been described as having cytotoxic effect toward several cell lines, DA1-3b/M2 (IC_50_ = 10.4 μM), K562 (IC_50_ = 25.1 μM), K562R and 697 (IC_50_ = 38 μM; Alkhatib et al., 2010).

### *In vivo* cytotoxicity

*In vitro* results can be taken as first clue for bioactivity, but they do not necessarily reflect activity in living organisms. Biotransformation in the liver degrades many natural products so that they finally lose activity *in vivo* (Reichling et al., 2009).

#### *Nigella sativa* and thymoquinone

The essential oil of *N. sativa* reduced solid tumor volume in mice in a dose-dependent manner (2.5 cm^3^ for untreated vs. 0.22 and 0.16 cm^3^ for 30 and 50 μL/mouse, respectively). In addition, it reduced cancer metastasis (14 for untreated mice vs. 2 and 0 for 30 and 50 μL/mouse, respectively) and improved survival of mice (83% mortality in untreated group vs. 16.6 and 0% for 30 and 50 μL/mouse, respectively; Ait Mbarek et al., 2007). Thymoquinone significantly decreases the tumor size and mass in a sarcoma 180-bearing mice xenograft model (Das et al., 2012). In addition, it inhibited angiogenesis as observed by a decrease of CD31 expression.

#### *Trigonella foenum-graecum* and diosgenin

An aqueous extract from the seeds of fenugreek slowed the progression of DMBA-induced breast cancer (40 vs. 80% incidence; Amin et al., 2005). Furthermore, it decreased the mean tumor number (2.0 vs. 3.5) and weight (3.0 g vs. 5.0 g) in rats. The histology was also markedly different with the extract-treated rats bearing only mild-to-moderate hyperplasia. Supplementation with 0.01% fenugreek seed powder for 8 weeks decreased total colonic aberrant crypt foci, while continuously feeding rats with fenugreek seed powder decreased multicrypt foci in azoxymethane-induced aberrant crypt foci formation (Raju et al., 2004). These effects were observed both in the initiation and promotion stages of carcinogenesis. Seed extract was given at pre-initiation, post-initiation, promotion, and throughout all stages to a DMBA+TPA-induced mouse skin carcinogenesis model (Chatterjee et al., 2012). The treatment decreased the cumulative papilloma count compared to control. Furthermore, it decreased cancer incidence and tumor burden. In addition, it decreased the latent period. A methanol extract reduced the number of mice bearing tumors (13 vs. 18 for untreated control), the tumor incidence (65 vs. 90% for untreated), the average number of tumors (102 vs. 221 for untreated control mice) and tumor multiplicity (7.84 vs. 12.27 for untreated) in a DMBA-TPA-induced skin carcinogenesis model (Ali et al., 2014). Pre-treatment of mice inoculated with Ehrlich ascites carcinoma (EAC) with an alcoholic extract of fenugreek decreased the number of EAC cell count per mouse (0.208 × 10^6^ at 200 mg/kg i.p. vs. 4.42 × 10^6^; Sur et al., 2001). Furthermore, post-treatment also decreased the EAC cell count per mouse (0.41 × 10^7^ at 200 mg/kg i.p. vs. 1.68 × 10^7^).

The supplementation with 0.1% diosgenin, the major compound of fenugreek, decreased both total colonic aberrant crypt foci and multicrypt foci in azoxymethane-induced aberrant crypt foci formation on initiation and promotion stages (Raju et al., 2004). Diosgenin is able to decrease tumor mass and size in mice bearing sarcoma 180 (Das et al., 2012). Furthermore, it decreased the expression of CD31, suggesting that diosgenin revealed antiangiogenic effects *in vivo*.

### Cell cycle perturbations

The effect of cytotoxic compounds is frequently measured by detection of cellular DNA with propidium iodide and subsequent measurement by flow cytometry. The resulting DNA histograms show the distribution of cell populations in the cell cycle. Anticancer compounds at subtoxic concentrations frequent led to an arrest of the cell cycle in the G1, S, or G2/M phase resembling cytostatic effects. Cytotoxicity of compounds results in cell death, which appears as dead cell fragments below the G0/G1 fraction. Hence, flow cytometric measurements can deliver data on both cytostatic and cytotoxic effects of anticancer substances. Cell cycle is a highly-regulated process by the sophisticated activity of cyclin-dependent kinases (CDKs). This type of kinases are activated by the presence of cyclins and de-activated by the presence of CDK inhibitors (Bassermann et al., 2014). The type of CDK-cyclin complex is dependent on extracellular signals as well as intrinsic information. The CDK inhibitors belong to two different families, the INK4 and CIP/KIP proteins. The first group interacts with CDK4/6 kinases inhibiting the capacity to bind to D-type cyclins, e.g., p16^INK4a^, while the latter binds to CDK-cyclin complexes, such p21^Cip1^ and p57^Kip2^ (Yun et al., 2013; Ruijtenberg and van den Heuvel, 2016). CDK inhibitors such as p21, p27, and p53 are tumor suppressor genes that block both cell proliferation and migration (Yun et al., 2013). Due to the importance of cell cycle progression for tumor development, several authors have described the cell cycle arrest capacity of plant extracts as well as isolated major compounds. In Figure 5 the effects of the extracts and major phytochemicals (thymoquinone, diosgenin and protodioscin) on cell cycle are represented.

#### Thymoquinone

To the best of our knowledge, no studies addressing the cell cycle arrest capacities of whole extracts or oils from *N. sativa* were conducted to the date. Thus, only studies with its major compound (thymoquinone) will be referred. Thymoquinone was able to cause cell cycle arrest at S phase (Khalife et al., 2016). By contrast, Woo et al. (2011) described that this compound caused accumulation of cells in the sub-G1 phase without effect on p53 activation. This compound caused cell cycle arrest at G1 after 24 h, but accumulated cells in the sub-G1 phase after 48 h treatment. Furthermore, thymoquinone induced the expression of p27 and p53, but downregulated the expression of cyclin D1 and E (Rajput et al., 2013). It caused the accumulation of sub-G1 cells (25.6 vs. 1.3% in untreated cells; Arafa et al., 2011). In p53-defective Jurkat lymphoblastic leukemia cells, the compound caused cell accumulation in sub-G1 (Alhosin et al., 2010). Gurung et al. (2010) described an accumulation in the sub-G1 phase in DNA-PKcs-normal (M059K) and -defective (M059J) glioblastoma cell lines. Thymoquinone also increased the sub-G1 fraction in U266 multiple myeloma cells (Li et al., 2010).

#### *Trigonella foenum-graecum* and diosgenin

A crude methanol extract of fenugreek seeds drove cells into the sub-G1 (~49.1%) and G1 phase (65% cells) in HepG2 (Khalil et al., 2015). In pancreatic cancer cells, fenugreek exerted two different effects. In both LNCaP and PC-3 cell lines, it increased accumulation of cells in sub-G1, while in PC-3 cells it also caused cell cycle arrest in the G2/M phase (Shabbeer et al., 2009). An ethanolic extract caused an increase of the sub-G1 fraction (30.1% at 100 μg/mL) as well as a cell cycle arrest at G2/M (Sebastian and Thampan, 2007). Diosgenin, a saponin isolated from fenugreek, caused sub-G1 accumulation after 48 h at 50 μM (51 vs. 9% for untreated cells; Shishodia and Aggarwal, 2006). Similarly, the treatment of squamous cell carcinomas with diosgenin caused an increase of the sub-G1 fraction (Das et al., 2012). The saponin causes cell cycle arrest at G2/M phase as soon as after 6 h of treatment (22.9 vs. 17.6%) due to an increase in the expression of p21, while after 24 h of treatment there was also an accumulation in the sub-G1 phase (Leger et al., 2004). After 36 h treatment diosgenin (20 μM) caused cell cycle arrest at G2/M with concomitant decrease in cyclin B1 and p21, and increase in cdc2 in K562 (Liu et al., 2005). In NB4 cells, diosgenin also caused arrest at the G2/M phase with an increase in p53 expression. Furthermore, after 48 h the arrest changed to sub-G1 phase accumulation simultaneously with DNA fragmentation. Diosgenin treatment up to 24 h, arrested cells in the G1 phase (50 vs. 35%) with an associated decrease of cells in the S phase (21 vs. 46%) in 1547 osteosarcoma cells (Moalic et al., 2001). At 48 h, a sub-G1 peak appeared. Concomitantly, an upregulation of p21 and p53 was observed.

#### Marrubium vulgare

An ethanolic extract of *M. vulgare* caused DNA fragmentation in a time-dependent manner (Paunovic et al., 2016) in B16 and U251 cell lines. Furthermore, it caused cell cycle arrest at the S and G2/M phases.

### Cell death induction

**Figure 6:**
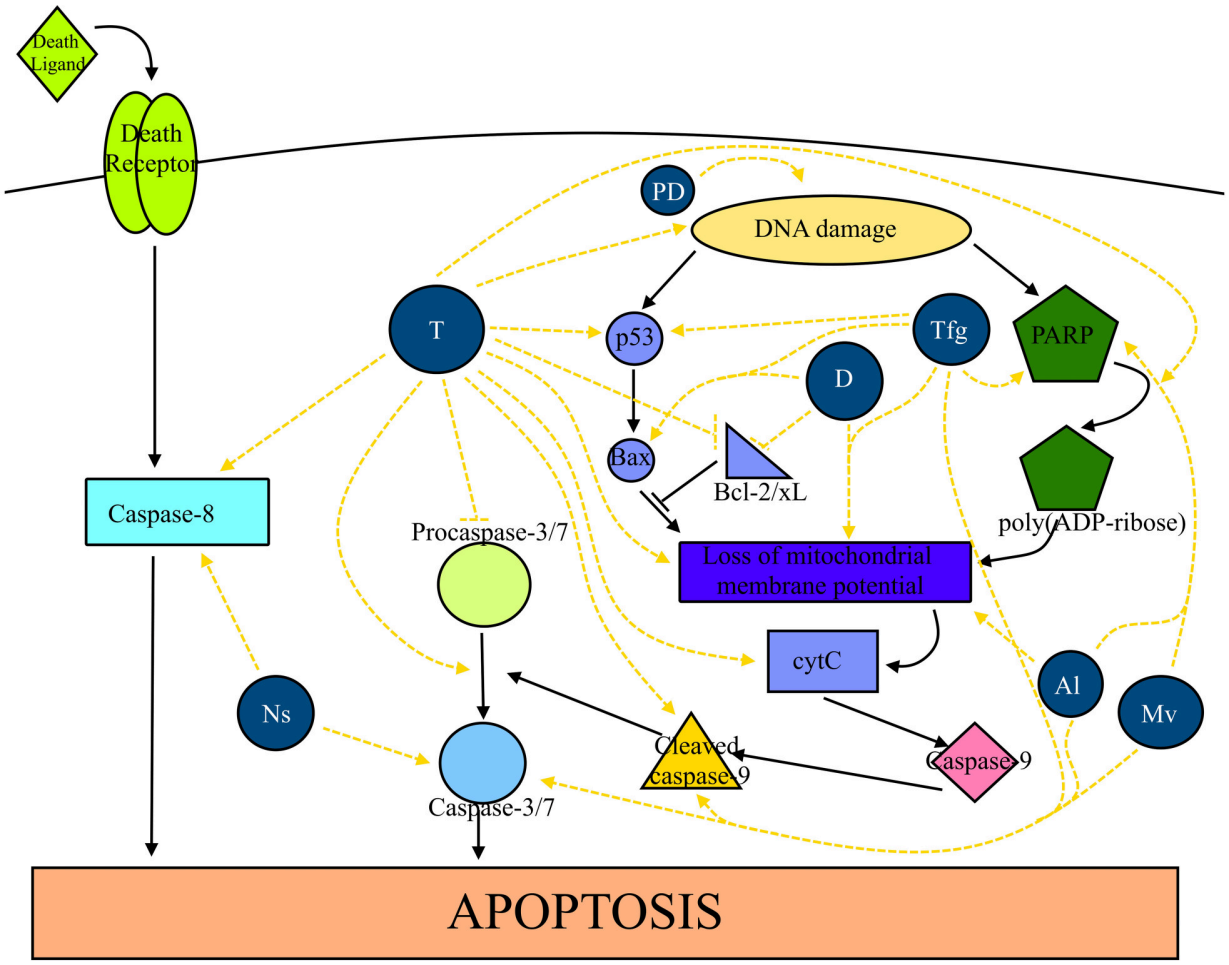
Plants traditionally used in cancer therapy in North Africa as well as their compounds are able to induce apoptosis in several cancer types. Extracts from *N. sativa* (Ns) promote the activity of caspase-8, -3, and -7. Thymoquinone (T) enhances the activity of caspase-8, the conversion of procaspase-3 in caspase-3, the activation of caspase-9, Bax, cleavage of PARP, release of cytochrome c (cytC), loss of mitochondrial membrane potential and causes DNA damage. Furthermore, it inhibits the activity of procaspase-3, Bcl-2 and Bcl-xL. *T. foenum-graecum* (Tfg) exerts a stimulatory effect on caspase-3/7, p53, and Bax and causes the loss of mitochondrial membrane potential and DNA damage. Diosgenin (D) causes loss of mitochondrial membrane potential and blocks the activity of Bcl-2 and Bcl-xL. Protodioscin (PD) cause DNA damage. Extracts from A. longa (Al) and *M. vulgare* (Mv) causes the activation of caspase-3/7 and the cleavage of caspase-9.

Apoptosis is a genetically programmed cell death with two main pathways, an intrinsic pathway characterized by the release of cytochrome c and posterior activation of caspase-9, and an extrinsic pathway characterized by the ligation of a death ligand (Walsh, 2014). While apoptosis controls whole cells, autophagy is a catabolic process in which intracellular organelles and macromolecules undergo destruction and recycling (Lapierre et al., 2015). In Figure 6 the effects of extracts from North African plants and isolated compounds (thymoquinone, diosgenin and protodioscin) in cell death by apoptosis are shown.

#### *Nigella sativa* and thymoquinone

A supercritical carbon dioxide extract obtained from the seeds of black cumin increased caspase-3 and-7 levels by 2- and 1.5-fold at 100 and 80 μg/mL, respectively, in MCF-7 breast cancer cells. At 100 μg/mL, it also increased caspase-8 levels by 2-fold. The extract increased the apoptotic index (5.65% untreated cells vs. 52.37% at 60 μg/mL; Baharetha et al., 2013). Thabrew et al. (2005) demonstrated that an aqueous extract inhibited DNA (IC_50_ = 3 mg/mL) and protein synthesis in HepG2 cells. A nanoemulsion of essential oil caused cell death by either apoptosis (~44% after 24 h) and necrosis (~28% after 24 h; Periasamy et al., 2016). Thymoquinone, the major compound of *N. sativa*, caused DNA fragmentation, induced apoptosis (36% vs. 2.5% after 48 h), induced the expression of caspase-8 precursor, and downregulated procaspases-3 (Khalife et al., 2016). Furthermore, decreased the levels of procaspase-8, -9 and -7 as well as the protein and mRNA levels of Bcl-2, Bcl-xL and survivin in a time-dependent manner while increasing the Bax/Bcl-2 ratio (Woo et al., 2011). Dastjerdi et al. (2016) suggested that thymoquinone induced apoptosis in MCF-7 cells in a p53- and time-dependent manner. In addition, this compound caused DNA fragmentation and morphological changes associated with apoptosis and disrupted the mitochondrial membrane potential, decreased the Bcl2/Bax ratio and activated caspases-3, -7, and -9 (Arafa et al., 2011). Furthermore, it triggered the release of cytochrome c from mitochondria, increased the expression of pro-apoptotic Bax proteins and downregulated the expression of the anti-apoptotic proteins Bcl2 and survivin (Rajput et al., 2013). Rooney and Ryan (2005) demonstrated that the proapoptotic effects are mediated by glutathione (GSH) depletion, since the pre-treatment with buthioninesulfoximine, a selective inhibitor of GSH, increased the number of apoptotic cells (31.7 vs. 55.1%). On the other hand, this effect was caspase-3-dependent, since the number of apoptotic cells decreased 4.9-fold after treatment with a caspase-3 inhibitor (41.1 vs. 8.4%). Kundu et al. (2014) reported that it induced apoptosis in HCT116 cells by decreasing the levels of Bcl-2 and Bcl-xL, but increasing Bax levels. Furthermore, it cleaved caspases-9, -7, -3 and PARP and activated caspase-3. Treating a neuroblastoma cell line, Neuro-2a, with thymoquinone downregulated the mRNA and protein levels of Bcl-2, upregulated the mRNA and protein levels of Bax, caused a loss of mitochondria membrane potential and triggered the release of cytochrome c to the cytosol. In addition, it activated caspases-3 and -9 with concomitant increase in PARP, while decreasing the protein levels of XIAP, a selective inhibitor for caspases-3, -7, and -9. All of them are signals that lead to cell death by apoptosis (Paramasivam et al., 2012). By contrast, the compound induced cell death by autophagy inhibition in glioblastoma cells lines (Racoma et al., 2013). The treatment increase LC3-II and p62 without changing the levels of Beclin-1. Concomitantly, vacuolization of cells, disruption of lysosomal membrane and modulation of lysosome location within the cell was observed. Furthermore, the compound activated cathepsin and induced caspase-independent cell death induction. In DNA-PKcs-normal and defective glioblastoma cell lines, the treatment triggered the release of cytochrome c to the cytosol, induced apoptotic cell death rather than necrosis, caused DNA damage, decreased telomerase activity and caused disruption of telomeres length maintenance. These effects were more predominant in DNA-PKcs-normal glioblastoma cells (Gurung et al., 2010). Li et al. (2010) described caspase-3-dependent apoptosis in U266 multiple myeloma cells. According to Zubair et al. (2013), thymoquinone-induced cell death in prostate cancer cell lines was mediated through ROS-induced DNA damage since the effect was inhibited in the presence of a copper-chelating agent (neocuproine).

#### *Trigonella foenum-graecum* and diosgenin

The treatment of several cancer cell lines (T-cell lymphoma, B-cell lymphoma, thyroid papillary carcinoma, and breast cancer) with an aqueous extract from the seeds of fenugreek induced apoptosis, if compared to the control (Alsemari et al., 2014). Similarly, a crude methanol extract triggered apoptosis by activating caspase-3 and up-regulated the expression of p53 and PCNA in HepG2 cells (Khalil et al., 2015). Furthermore, the expression of Bax and the cleavage of PARP were also increased. In DU-123 cancer cells, fenugreek downregulated the expression of mutant p53 (Shabbeer et al., 2009). On the other hand, the extract inhibited the phosphorylation of EGFR in PC-3 cancer cells. A methanol extract of the whole plant induced apoptosis in MCF-7 cells (46.1 and 58.9% apoptotic cells after 24 and 48 h, respectively; Alshatwi et al., 2013). Furthermore, it increased the expression of apoptotic genes, 0.9-fold increase in *caspase-3*, 0.25-fold in *caspase-8*, 0.3-fold in *caspase-9*, 1.7-fold in *p53*, 8.8-fold in *fas*, 0.12-fold in *FADD*, 0.4-fold in *bax* and 0.7-fold in *bak*. These results show that fenugreek induced apoptosis *via* a fas-dependent pathway, however, independent of FADD, Bax and Bak. A chloroform extract induces apoptosis in MCF-7 cells in a dose- and time-dependent manner (23.2% and 73.8% apoptotic cells after 24 and 48 h, respectively, with 50 μg/mL; Khoja et al., 2011). Treating MCF-7 cells with an ethanolic extract induced apoptosis as observed by the flipping of phosphatidylserine from the inner to the outer phospholipid bilayer, loss of mitochondrial membrane potential, and DNA fragmentation (Sebastian and Thampan, 2007). In addition, the treatment increased the expression of apoptotic genes several folds, e.g., *caspase-3* (3.5-fold), *caspase-8* (5.5-fold), *caspase-9* (3.7-fold), *p53* (1.4-fold), *fas* (1.7-fold), *FADD* (2.6-fold), *bax* (3.2-fold), and *bak* (4.4-fold). Treatment of Jurkat leukemia cells with an ethanolic extract induced the formation of vacuoles followed by cell membrane disintegration with a concomitant increase of LC-3 expression, all of which are signals of autophagic cell death (Al-Daghri et al., 2012). The treatment of DMBA-TPA-induced tumors in mice with a methanol extract of fenugreek decreased the number of PCNA-positive nuclei and increased the expression of p53 (Ali et al., 2014).

Diosgenin induced apoptosis in HT-29 colon cancer cells by decreasing Bcl-2 expression, while increasing the expression of caspase-3 (Raju et al., 2004). It induced apoptotic cell death as found by morphological and histological changes as well as chromatin condensation in squamous cell carcinomas. In addition, it decreased procaspase-3 levels, decreased Bcl-2 levels, increased Bax levels and inhibited the phosphorylation of JNK and Akt (Das et al., 2012). In A549 lung cancer cells, diosgenin decreased the mRNA levels of hTERT (Rahmati-Yamchi et al., 2014). This saponin disrupted mitochondrial membrane potential as well as the intracellular calcium concentration (578 vs. 210 nM after 12 h; Leger et al., 2004). Furthermore, it increased the Bax/Bcl-2 ratio, caused PARP cleavage with concomitant DNA fragmentation. The increase in intracellular calcium caused a translocation to the membrane and subsequent cPLA_2_ activation. In K562 leukemia cells, it also increased the intracellular calcium concentrations, hyperpolarization of mitochondrial membranes after 24 h followed by depolarization after 48 h (Liu et al., 2005). In addition, it activated caspase-3, but decreased the expression of Bcl-2 and Bcl-xL and increased Bax expression. Diosgenin induced apoptosis with an increase in hsp70 mRNA expression and in Bax/Bcl-2 ratio (Moalic et al., 2001).

Protodioscin induced apoptosis in HL-60 leukemic cells with typical morphological changes associated with apoptosis (increased numbers of apoptotic bodies, number of cells in the hypodiploid phase and DNA fragmentation; Hibasami et al., 2003).

#### Aristolochia longa

The treatment of BL41 Burkitt's lymphoma with an aqueous extract from the roots induced apoptosis (20.7 vs. 2.7%; Benarba et al., 2012) with concomitant mitochondrial membrane potential loss (53 vs. 11.6%). Furthermore, it activated caspases-3 and -9 followed by PARP cleavage without affecting the activity of caspase-8. This suggests that *A. longa* activated the intrinsic pathway of apoptosis.

#### Marrubium vulgare

Treating B16 and U251 cells with an ethanolic extract of *M. vulgare* cause increased fractions of both early and late apoptotic cells (Paunovic et al., 2016). In addition, it activated caspases-9 and -3 with concomitant PARP cleavage. A loss of mitochondrial membrane potential was also observed in both cell lines.

### Signal transduction pathways

**Figure 7:**
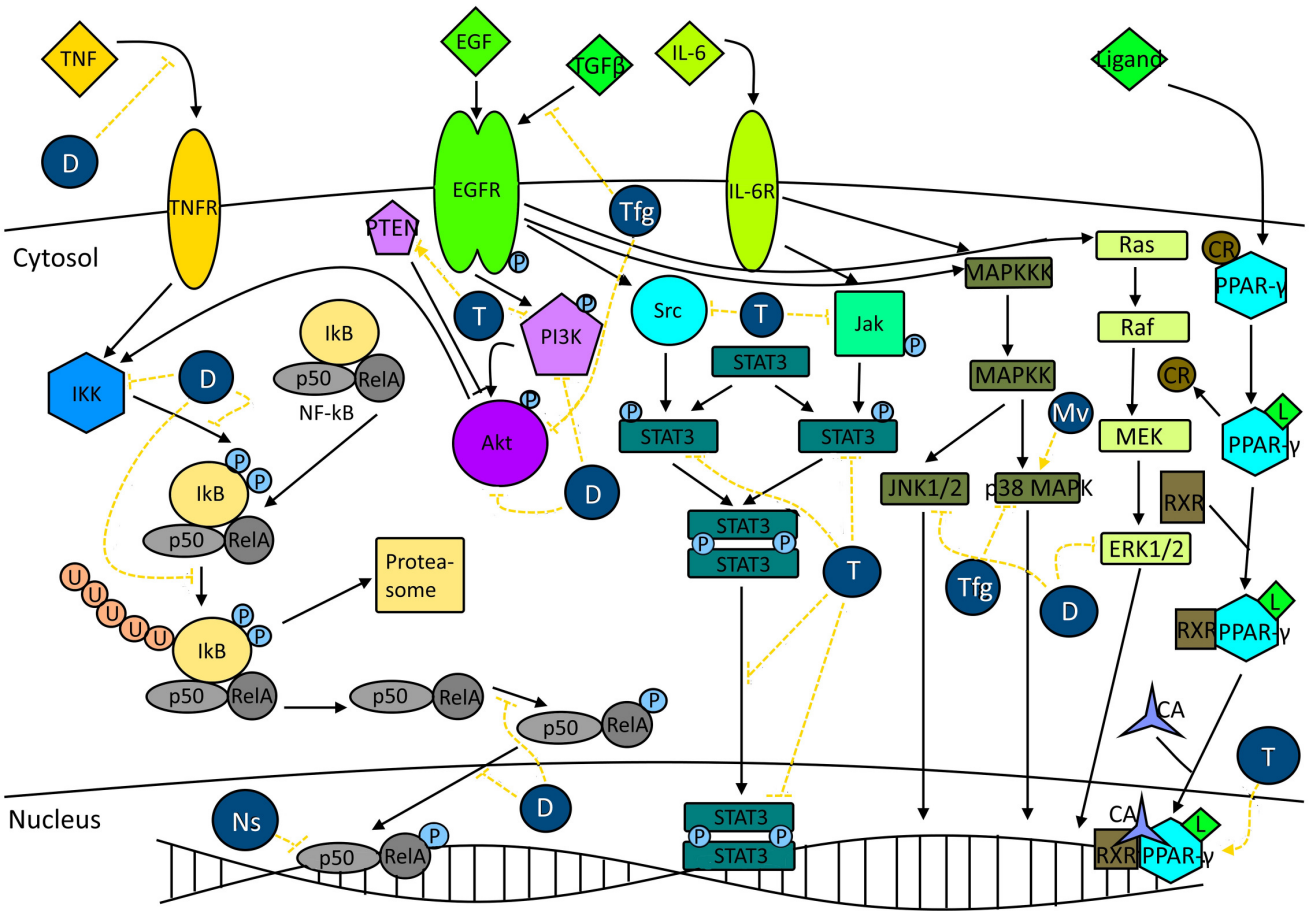
Plants from the North Africa and major compounds are able to modulate signal transduction pathways associated with cancer survival. *N. sativa* extracts (Nv) are able to decrease NF-κB activity. The major compound, thymoquinone (T) decreases STAT3 phosphorylation, nuclear translocation and STAT3-induced gene expression. Furthermore, it inhibits or activates the activity of PTEN, inhibits the phosphorylation of PI3K and promotes the activity of PPAR-γ. Extracts from *T. foenum graecum* (Tfg) exert an inhibitory effect on the phosphorylation of EGFR induced by TGFβ as well as Akt phosphorylation. In addition, it decreases the activity of p38 MAPK. Diosgenin (D) inhibits NF-κB activity induced by TNF, inhibits IKK (IkBα kinase) activity and decreases phosphorylation and degradation of IkBα. Furthermore, it decreases the phosphorylation and nuclear translocation of p65 (RelA). In addition, it inhibits the phosphorylation of PI3K, Akt, ERK1/2, and JNK1/2. Extracts from *M. vulgare* (Mv) exert a stimulatory effect on p38 MAPK activity. CR, Co-repressor; CA, Co-activator.

The cellular metabolism is tightly controlled by several pathways. In cancer cells, some of those pathways are dysregulated. NF-κB has been associated with the regulation of several processes such as inflammation, cell growth, and apoptosis (Ghosh and Dass, 2016). This nuclear transcription factor also controls several other biological pathways such as the MAPK or PI3K/Akt pathways. The activation of NF-κB is associated with increased expression of several genes whose products are associated with tumorigenesis, such as antiapoptotic proteins (e.g., survivin, Bcl-2), COX-2, matrix metalloproteinase-9, iNOS, and cell cycle regulation proteins (e.g., cyclin D1; Shishodia and Aggarwal, 2006). Another dysregulated pathway is STAT3. This signal transduction pathway has been associated with cell proliferation, cell survival and angiogenesis (Aggarwal et al., 2009; Becker et al., 2014; Yamamoto et al., 2014). STAT3 is activated by several intrinsic and extrinsic factors such as IL-6, JAKs, ERK, cigarette smoke (Aggarwal et al., 2009). In normal cells, this pathway is only transiently activated and tightly regulated. However, it is constitutively activated in cancer cells (Kortylewski et al., 2005). While NF-κB and STAT3 pathways are activated in cancer cells, PPAR pathway is inactivated, because this pathway negatively controls proliferation and survival (Reka et al., 2011). Furthermore, PPAR inhibits NF-κB and STAT3 pathways. In addition, this pathway is also associated with cell cycle arrest and apoptosis induction (Dicitore et al., 2014). The effects of the extracts and major compounds (thymoquinone and diosgenin) on signal transduction pathways are represented in Figure 7.

#### *Nigella sativa* and thymoquinone

Seed oil obtained from non-heated seeds of *N. sativa* inhibited NF-κB activity by around 50% while heating the seeds to 50°C caused a total inhibition of NF-κB in MC38 colon carcinoma cell line (Agbaria et al., 2015). The major compound of black cumin, thymoquinone, acts on several signal transduction pathways, such as PPAR-γ and STAT3. In fact, thymoquinone induced the expression of PPAR-β/δ (unspecifically) and PPAR-γ (specifically) without significant effect on PPAR-α in MCF7 cells. The increase of PPAR-γ activity was dose- and time-dependent (Woo et al., 2011). Furthermore, thymoquinone inhibited the activation of PI3K/Akt signaling pathway, decreased the phosphorylation of the PTEN and PDK1, the expression of phospho-GSK-3β and Bad, increased cleavage of caspase-9, increased the activity of GSK-3β, Bad and caspase-9 and downregulated the expression of Akt downstream of the mTOR-dependent translational machinery (Rajput et al., 2013). In addition, in a doxorubicin-resistant MCF-7/Dox cell line, thymoquinone caused PARP cleavage with concomitant PTEN upregulation and increase of downstream proteins (Arafa et al., 2011). In p53-defective Jurkat lymphoblastic leukemia cells, the treatment increased both p73 α and β expression, while decreasing the expression of UHRF-1, DNMT1 and HDAC1 in a dose-dependent manner. In HCT116 cells, it decreased the phosphorylation of STAT3, the nuclear localization of p-STAT3 and the expression of STAT3 target genes related to survival (e.g., survivin and cyclin-D1 and -D2), while increasing the expression of cell cycle regulatory proteins (e.g., p27). Furthermore, thymoquinone inactivated kinases responsible for the phosphorylation of STAT3, EGFR tyrosine kinase, JAK2 and Src (Kundu et al., 2014). The compound also modulated the STAT3 pathway in U266 multiple myeloma cells. Thymoquinone inhibited the phosphorylation of constitutively expressed STAT3 and depleted the nuclear translocation of STAT3 without neither affecting protein levels of STAT3 nor the phosphorylation and protein levels of STAT5 (Li et al., 2010). Furthermore, thymoquinone suppressed IL-6-induced phosphorylation of STAT3, activation of Akt and the expression of STAT3-dependent genes. In addition, it inhibited the phosphorylation of constitutively expressed JAK2 and of activated protein tyrosine phosphatases and it induced the expression of SH-PTP2, which negatively regulates STAT3 activation. Finally, thymoquinone downregulated the expression of STAT3-dependent genes associated with cell survival, proliferation and angiogenesis.

#### *Trigonella foenum-graecum* and diosgenin

A fenugreek extract decreased the phosphorylation of EGFR induced by TGFβ in PC-3 cancer cells (Shabbeer et al., 2009). Furthermore, it inhibited the TGFβ-induced Akt phosphorylation and induced p21 expression. In an animal model of skin carcinogenesis induced by DMBA-TPA, a methanol extract decreased p38MAPK expression (Ali et al., 2014). Diosgenin inhibited TNF-induced expression of NF-κB on KBM-5 cells in a dose- and time-dependent manner (Shishodia and Aggarwal, 2006). Furthermore, it decreased the degradation and phosphorylation of IκBα and the activation of IκBα kinase. In addition, it inhibited the phosphorylation and subsequent p65 nuclear translocation and activation of the Akt pathway. Concomitantly, the saponin inhibited the expression of NF-κB-dependent genes, e.g., antiapoptotic genes, MMP-9 and cyclin-D1. These effects were found both in ER-positive and ER-negative cells, thus suggesting that NF-κB inhibition was ER-independent. Diosgenin inhibited the phosphorylation of PI3K, Akt, ERK1/2, JNK1/2 in a dose- and time-dependent manner (Chen et al., 2011). Furthermore, NF-κB nuclear translocation was inhibited in the presence of diosgenin. Diosgenin treatment activated NF-κB in 1547 osteosarcoma cells (Moalic et al., 2001).

#### Marrubium vulgare

Paunovic et al. (2016) described the activation of p38 MAPK with concomitant activation of proapoptotic genes and cell cycle inhibitor and inhibition of antiapoptotic genes after treatment with an ethanolic extract. Furthermore, it conversed LC3-I to the LC3-II in both B16 and U251 cell lines with associated upregulation of autophagy-associated genes.

### Invasiveness, migration, and metastasis

**Figure 8:**
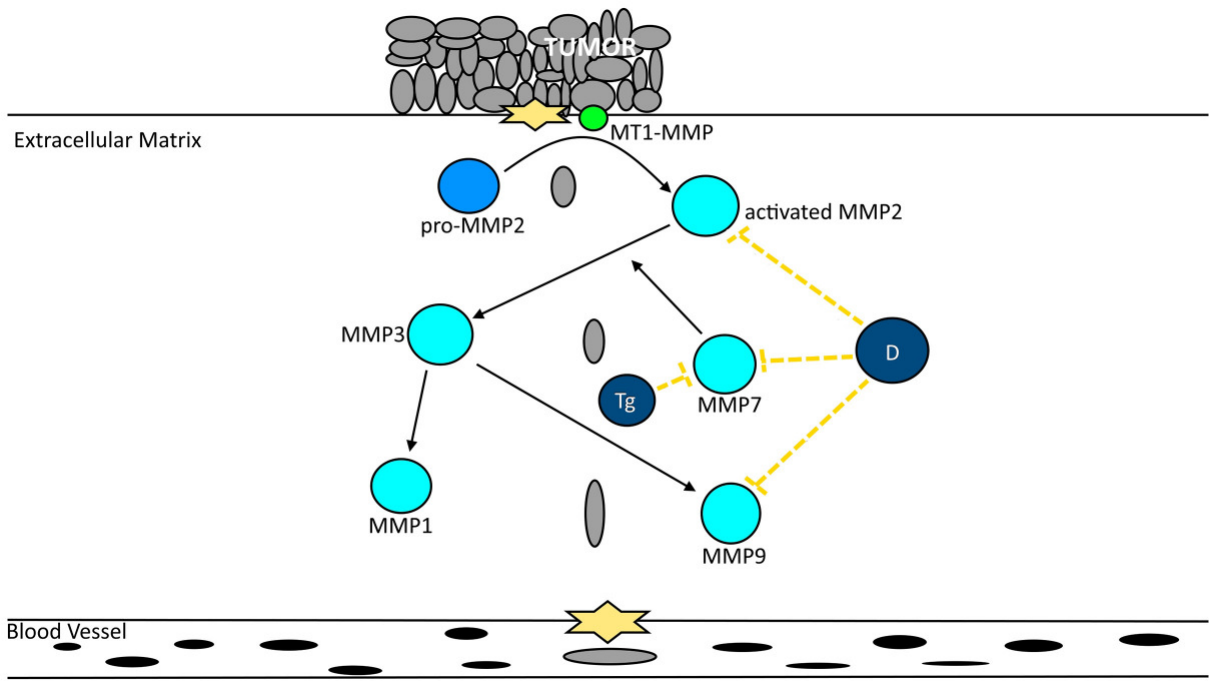
Extracts from North African plants and major compounds decrease cancer migration by inactivating metalloproteinases. Diosgenin (D) decreases the activity of MMP-2, -7, and 9. Trigonelline (Tg) decreases the activity of MMP-7.

Cancer cell invasion is one of the major challenges to achieve relapse-free and sustainable patient survival (Alexander and Friedl, 2012). Tumor invasion is characterized by cell motility, migration and degradation of the extracellular matrix (Domoto et al., 2016) with concomitant intravasation into blood and/or lymphatic vessels and metastasis of distant organs (Alexander and Friedl, 2012). Although, only very few migrant cells initiate *de novo* tumor growth in a new organ (Oh et al., 2015), metastasis is responsible for most of the cancer-related deaths. Invasion is regulated by several factors (Alexander and Friedl, 2012), such as the PTEN/PI3K and MAPK/ERK pathways as well as increased MMP expression and activity. The effect of the extracts and major compounds on the major metalloproteinases associated with cancer invasiveness can be found in Figure 8.

#### *Nigella sativa* and thymoquinone

A supercritical carbon dioxide extract inhibited colony formation in a dose-dependent manner, decreased cell motility (63.22% inhibition at 40 μg/mL) and cell invasion (79.29% inhibition at 40 μg/mL; Baharetha et al., 2013). Thymoquinone inhibited migration of both MCF-7 and MDA-MB-231 cells and invasion of MDA-MB-231 cells (Woo et al., 2011).

#### Diosgenin and trigonelline

To the best of our knowledge, no studies assessing the anti-invasiveness and anti-migration capacities of whole extracts from *T. foenum-graecum* have been carried out as of yet. Therefore, we only focus on results related to diosgenin. Treatment with diosgenin inhibited TNF-induced invasion in H1299 cells by 50% and ~40%, respectively, prior and after invasion induction (Shishodia and Aggarwal, 2006). Non-cytotoxic doses decreased the migration of PC-3 cells in a time-dependent manner as well as the cell invasion in a dose-dependent manner (22 and 40% invasion inhibition at 10 and 20 μM, respectively; Chen et al., 2011). Furthermore, the saponin decreased MMP-2 and MMP-9 activity with concomitant suppression of the mRNA and protein expression. In addition, it also decreased the mRNA levels of MMP-7 and EMMPRIN (extracellular inducer of matrix metalloproteinase), while increasing the levels of TIMP-2 (tissue inhibitor of metalloproteinase-2). Tube formation in HUVECs induced by conditioned medium by PC-3 cells was inhibited suggesting that diosgenin might inhibit angiogenesis.

Trigonelline (Figure 4, 4), the major alkaloid of fenugreek has been described to act in an anticarcinogenic manner (El Bairi et al., 2017). In fact, Hirakawa et al. (2005) demonstrated that the ROS-induced migration of AH109A hepatoma cells was inhibited in a dose-dependent manner by this alkaloid. Furthermore, this compound decreased the motility of Hep3B (Liao et al., 2015) as well as the expression of MMP-7, the expression of phospho-Nrf2, PKCα, ERK1/2, and p38, while increasing the phosphorylation of Raf on serine-259. In addition, trigonelline decreased the expression and activity of several anti-oxidative enzymes.

## Toxicological evidence

Toxicity testing represents an important step in the drug development process evaluating the potential of a medicinal plant, before it can be further considered for clinical trials.

### *Nigella sativa* and thymoquinone

Black cumin is widely used in traditional medicine. However, it is commonly used in low doses due to the lack of scientific evidence regarding its toxicity (Al-Ali et al., 2008; Dollah et al., 2013). Nevertheless, some authors described toxic effects of *N. sativa* both *in vitro* and *in vivo*. In fact, Khader et al. (2007) described that 0.3 mg/mL of a seed extract from *N. sativa* exerted genotoxicity since it promoted the formation of MNNG-induced chromosomal aberrations and the appearance of micronuclei. The same authors described that thymoquinone (20 μM) decreased cell proliferation, while necrosis was augmented at concentrations as low as 2.5 μM. Higher concentrations exerted acute cytotoxicity (Khader et al., 2009). Regarding the genotoxicity of thymoquinone, the number of chromosomal aberrations and micronuclei increased in a dose-dependent manner up to 10 μM. *In vitro* assays are of limited predictive power regarding potential toxicity in human subjects due to the fact that cell cultures do not reflect the biotransformation that occurs *in vivo* (Reichling et al., 2009). The LD_50_ of a fixed oil from the seeds of *N. sativa* was determined for its acute and chronic toxicity (Zaoui et al., 2002). For acute toxicity, the LD_50_ was 28.8 and 2.06 mL/kg body weight of mice for oral and intraperitoneal administration, respectively. The LD_50_ of an alcoholic extract administered intraperitoneally was 561 mg/kg (Paarakh, 2010). The acute toxicity of thymoquinone, the major constituent of *N. sativa*, was determined by Badary et al. (1998). The LD_50_ was 2.4 g/kg. High doses of thymoquinone induced hypoactivity and breathing difficulties before death. After 24 h of treatment, high doses of thymoquinone decreased liver, plasma and heart GSH levels. Furthermore, the activities of ALP, LDH and CPK, and plasma concentration of urea and creatinine were elevated. The chronic toxicity was determined in rats by giving 2 mL/body weigh/day for 12 weeks (Zaoui et al., 2002). At the end of the treatment, no differences in key enzymes were detected, serum cholesterol, triglycerides, and glucose significantly decreased. Furthermore, the counts for leukocytes and platelets decreased while hematocrit and hemoglobin increased. The treated groups presented a lower body weight at 6 weeks and onwards, whereas the organ weight remained unchanged. The subchronic administration of 30–90 mg/kg/day thymoquinone for 90 days showed no effect on key enzymes, body weight, hematological parameters nor did it cause histopathological changes (Badary et al., 1998). The oral administration of aqueous, methanol and chloroform extracts exerted no mortality 7 days post-treatment. However, at 21 g/kg of aqueous and chloroform extracts lowered the body weight (Vahdati-Mashhadian et al., 2005). The subacute effects of these extracts were determined by giving orally 2 g/kg/day for 14 days. At the end of the treatment, the aqueous extract decreased alkaline phosphatase (ALP), if compared to the control. Other enzymes were not affected. The chloroform extract decreased the activity of all tested enzymes. The oral intake of either fixed oil or essential oil was considered as safe, since no changes on serological indexes and white and red cell count appeared even 56 days after treatment (Tauseef Sultan et al., 2009). Al-Ali et al. (2008) demonstrated that the toxicity of *N. sativa* depends on the type of administration. Intraperitoneal injections to mice and rats demonstrated low LD_50_ values (104.7 and 57.5 mg/kg for mice and rats, respectively), whereas upon oral administration the lethal doses were 870.9 and 794.3 mg/kg, respectively. Furthermore, the intraperitoneal administration led to abdominal muscle contraction and ataxia, which persisted for a couple of hours. After 6 h, the animals were drowsy and less responsive. For the oral administration, the drowsiness and poor responsiveness was more gradual and lasted until the animal's death or vanished after 24 h. The oral administration of grounded seeds to rats in doses of 0.01, 0.10, and 1.00 g/kg body weight for 5 weeks caused no changes in urea and creatinine levels, neither did it cause histopathological changes in the kidney (Dollah et al., 2013). In addition to both *in vitro* and *in vivo* studies, several clinical trials have been conducted using either *N. sativa* and thymoquinone without demonstrating any severe side effects (Qidwai et al., 2009; Akhondian et al., 2011; Amin et al., 2015; Mohtashami et al., 2015). In addition to the rather safe toxicological profile, i.p. pre-treatment with 12.5 mg/kg. of *N. sativa* volatile oil decreased the hepatotoxicity induced by CCl_4_ by decreasing the level of serum enzymes and MDA content (Mansour et al., 2001). On the other hand, thymoquinone decreased cisplatin-induced nephrotoxicity by lowering urea and creatinine levels and improvement of polyuria, kidney weight, and creatinine clearance (Badary et al., 1997).

### *Trigonella foenum-graecum* and diosgenin

Fenugreek has been widely used in traditional medicine for treating several ailments and diseases, although several side-effects have been reported (Al-Ashban et al., 2010), including anti-fertility and abortifacient effects (Ouzir et al., 2016). The lethal dose of an alcoholic extract of fenugreek seed was above 1 g/kg i.p. (Sur et al., 2001). The intraperitoneal administration of an aqueous extract yielded an LD_50_ close to 4,000 mg/kg (Javan et al., 1997). The LD_50_ upon i.p. administration of an aqueous extract from the leaves of *T. foenum-graecum* was 1.9 g/kg, while the LD_50_ increased to 10 g/kg upon oral administration (Abdel-Barry et al., 1997). The i.p. administration of a glycoside extract from fenugreek revealed an LD_50_ of 0.65 g/kg, while the LD_50_ value increased to 7 g/kg upon oral administration (Abdel-Barry and Al-Hakiem, 2000). A fenugreek extract enriched in furostanolic saponins (>60% w/w) did neither show acute toxicity (LD_50_ > 5,000 mg/kg) nor sub-chronic toxicity (Swaroop et al., 2014). Supplementation with 100 mg/kg/day in drinking water for 3 months did not show alarming signs of chronic or acute toxicity (Al-Ashban et al., 2010). Similarly, intragastrical administration of fenugreek extract did not exert acute toxicity upon oral supplementation (Muralidhara et al., 1999). Although, several studies have demonstrated low toxicity of fenugreek extracts, some authors have established that these extracts are toxic to the fetus development (Khalki et al., 2010; Mozaffari et al., 2010). Furthermore, Khader et al. (2007) also described the mutagenic potential of *T. foenum-graecum*. Several clinical trials have been conducted using fenugreek, which described mostly non-serious side effects. In fact, the supplementation of diabetic individuals with 25 g/day for 24 weeks revealed a few side effects (diarrhea and excess flatulence) that subdued after a few days (Sharma et al., 1996). In a single-blind trial, 20 healthy men treated with an aqueous extract minimal side effects were observed (feeling of hunger, dizziness and frequency of micturition; Abdel-Barry et al., 1997). A double-blind, randomized and placebo-controlled 6-week trial in 60 healthy adult males showed no adverse side effects using a standardized fenugreek extract, except for three individuals that complained about slight stomach discomfort after taking the extract in the absence of food (Steels et al., 2011). In another double-blind, randomized, placebo-controlled three-period cross-over trial, 25 healthy male individuals were supplemented with fenugreek for three 14-day treatment period with a 14-day washout period, only few side effects were described (abdominal pain and urine smell; Chevassus et al., 2009). In a double-blind and placebo-controlled, Gupta et al. (2001) described that treatment with 1 mg/day of a hydroalcoholic seed extract for 2 months caused dyspepsia and mild abdominal distension without affecting the kidney and liver. A double-blind, randomized and placebo-controlled trial on 120 healthy adult male using a standardized seed extract showed no adverse effects during 12 weeks of treatment (Rao et al., 2016). Some minor side effects were observed that were dispersed between both treatment and control groups. Thus, they cannot be associated with fenugreek consumption. Swaroop et al. (2015) conducted an open label, one-arm, non-randomized, and post-marketing surveillance in 50 premenopausal women and did not find hepatotoxicity, nephrotoxicity and cardiotoxicity during the 90 days observation period. Furthermore, changes in the hemogram and total leukocyte count were not observed, although the hemoglobin levels slightly increased. A short-term, single site, double-blind, randomized and placebo-controlled study was carried out in 80 women, who consumed 600 mg/day of a standardized seed extract (Rao et al., 2015). No major adverse effects were observed with only two patients manifesting minor side effects in the treatment group.

### Aristolochia longa

Although beneficial effects of *A. longa* were reported not only against cancer, but also against several other ailments, this plant is rich in aristolochic acids (Cherif et al., 2014). These acids are responsible for the toxicity associated with many species of the *Aristolochia* genus (Yamani et al., 2015) and cause a syndrome named aristolochic acid nephropathy (Benzakour et al., 2012; Cherif et al., 2014). By contrast, several studies have assessed that extracts from the roots of *A. longa* to be safe in a single dose treatment (Benzakour et al., 2011; Benarba et al., 2012, 2016; Cherif et al., 2014). On the other hand, Cherif et al. (2014) described 1/6 mortality upon application of 4 g/kg and 2/6 mortality upon 5 g/kg. Low doses failed to exhibit sings of sub-chronic toxicity (Benzakour et al., 2011; Cherif et al., 2014). By contrast, high doses demonstrated serious toxicity, such as atypical locomotion, anorexia, asthenia, ataxia, diarrhea, and urination (Benzakour et al., 2011; Cherif et al., 2014).

## Scientific evidence on medicinal plants not traditionally used for cancer treatment

Several plants are widely used as by herbalists in North Africa to treat diseases other than cancer. Nevertheless, several studies have been conducted on some of those plants highlighting their potential as anticancer agents. Therefore, we also report on these medicinal plants. Diethyl ether and petroleum ether extracts from *Alhagi maurorum* demonstrated a comparable cytotoxicity toward several cancer lines, including C32 cells as the most susceptible to the first extract (IC_50_ = 2.7 μg/mL) and HeLa cells as the most susceptible to the latter (IC_50_ = 40.1 μg/mL; Loizzo et al., 2014). Methanolic extracts from *Artemisia herba-alba, Ruta chalepensis*, and *Peganum harmala* all demonstrated cytotoxic effects in a dose-dependent manner (Khlifi et al., 2013). An ethyl acetate extract of *Argania spinosa* inhibited the growth of MCF-7 cells (IC_50_ = 42 μg/mL; El Babili et al., 2010). Extracts of *Inula viscosa* were cytotoxic toward several cancer cell lines, including multidrug-resistant lines, with the hexane extract demonstrating the most promising activity (IC_50_ = 9.56–15.67 μg/mL; Merghoub et al., 2016). In a comparative study with 16 plants, the methanol extracts from *I. viscosa* and *Ononis hirta* were the most potent ones against all tested cancer cell lines. For instance, in MCF7 cells the IC_50_ values were in a range from 15.78 to 27.96 μg/mL (Talib and Mahasneh, 2010). A crude alkaloid extract from *Glaucium flavum* inhibited the growth of several human cancer cell lines in a dose dependent manner (IC_50_ = 7.9–13.6 μg/mL; Bournine et al., 2013a). Furthermore, this type of extract significantly inhibited the growth of MDA-MB-231 cells in a dose-dependent manner (Bournine et al., 2013b). Treating mice bearing Ehrlich ascites tumor with an methanolic extract of *Calligonum comosum* decreased the number of viable cells (99.2 × 10^6^ vs. 192.8 × 10^6^; Badria et al., 2007). In another study, a methanolic extract of *C. comosum* inhibited the viability of HepG2 carcinoma cells in a dose-dependent manner (IC_50_ = 9.60 μg/mL; Shalabi et al., 2015). Similarly, *Aloe vera* also decreased cell viability of HepG2 in a dose-dependent manner (IC_50_ = 10.45 μg/mL). The aqueous extract obtained from garlic (*Allium sativum*) decreased the viability of murine CT26. WT colon carcinoma cells (Lee et al., 2013). Several extracts from mulberry (*Morus alba*) decreased the viability of HepG2 in a time-dependent manner (Fathy et al., 2013). The essential oil of *Artemisia campestris* inhibited the growth of HT-29 cells (IC_50_ = 46.82 μg/mL; Akrout et al., 2011). Several extracts of *Thymelaea hirsuta* only weakly inhibited cell growth at high concentrations of 100 μg/mL (58.19–65.54% inhibition). Several roots extracts from *Linum usitatissimum* significantly inhibited the growth of Jeg3 trophoblast tumor cells (Abarzua et al., 2007). Infusions from *Mentha* spp., *Rosmarinus officinalis* and *Origanum majorana* inhibited the growth of several cancer cell lines, including HeLa, Jurkat and MCF-7 (Elansary and Mahmoud, 2015). Seed oil isolated from *Ecbalium elaterium* inhibited the growth of HT-29 colonic adenocarcinoma (IC_50_ = 4.86 μg/mL) and HT-1080 colonic fibrosarcoma cells (IC_50_ = 4.16 μg/mL; Touihri et al., 2015). Treating MCF-7 and OVCAR cells with 50 mg/L of hydromethanolic extract from the flower buds of *Cistus salviifolius* inhibited the growth by 35.16 and 36.85%, respectively (El Euch et al., 2015). The methanolic extract of *Achillea odorata* (50 μg/mL) inhibited the MCF7 cell growth of MCF-7, Hep2 and WEHI cancer cells in a dose-dependent manner with 42.90, 61.54, and 81.13% inhibition, respectively (Boutennoun et al., 2017). The growth of B16F10 was inhibited in a dose- and time-dependent manner (IC_50_ ~736 μg/mL after 24 h and ~650 μg/mL after 48 h) after treatment with an aqueous extract from the gall of *Limoniastrum guyonianum* (Krifa et al., 2014). Furthermore, it also decreased both tumor weight and size in B16F10-bearing mice. In a similar study, the same type of extract exerted antiproliferative activity toward HeLa cells in a dose- and time-dependent manner (IC_50_ = 170 and 140 μg/mL after 24 and 48 h, respectively; Krifa et al., 2013). A hexane extract from the bark of *Ficus drupacea* decreased the growth of several cancer lines with T24 cells as the most susceptible ones (IC_50_ = 21.32 μg/mL; Yessoufou et al., 2015). The anticancer effects of a methanolic extract of *Cleome arabica* have been described against several cell lines. SK-N-BE neuroblastoma cells were the most sensitives ones to this extract (Tigrine et al., 2013). Saffron (*Crocus sativus*) exerted antiproliferative activity against two aggressive prostate cancer cell lines injected into mice by decreasing tumor weights (Festuccia et al., 2014). Furthermore, it decreased the metastatic capabilities of the tumors. In another study, saffron extracts inhibited the growth of both p53^+/+^ and p53^−/−^ HCT116 cell lines in a dose- and time-dependent manner (Bajbouj et al., 2012).

In a MNNG-induced gastric carcinogenesis model, treatment with an aqueous extract from *C. sativus* inhibited the progression to the adenoma stage in a dose-dependent manner (Bathaie et al., 2013). Saffron also inhibited the growth of A549 lung cancer cells in a dose- and time-dependent manner (IC_50_ = 380 and 170 μg/mL after 48 and 72 h; Samarghandian et al., 2013). Dichloromethane and methanol extracts from *Limonium densiflorum* were cytotoxic against different cancer cell lines being the dichloromethane extract the most promising one (Medini et al., 2015). The ethyl acetate extract from *Cyperus rotundus* weakly inhibited the proliferation of K562 erythroleukemia cells (IC_50_ = 100 μg/mL; Kilani-Jaziri et al., 2009). This type of extract only weakly inhibited the growth of L1210 cell line (IC_50_ = 200 μg/mL), while a lyophilized infusion and a methanol extract exerted negligible activity (Kilani et al., 2008). Several extracts from the leaves of *Punica granatum* were screened for their antiproliferative effects toward MCF-7 cells (Bekir et al., 2013). The methanol extract was the most effective (IC_50_ = 31 mg/L). A hydroethanolic extract from the grains of *Echinochloa crus-galli* demonstrated potent cytotoxicity against several cancer cell lines (El Molla et al., 2016). Extracts from *Ceratonia siliqua* and *Quercus ilex* inhibited the proliferation of U87 glioblastoma in a dose-dependent manner (Amessis-Ouchemoukh et al., 2017). The growth of KB cancer cell line was inhibited in a dose-dependent manner by an aqueous extract from *Moringa oleifera* leaves (Sreelatha et al., 2011). Several extraction techniques from the leaves of various olive cultivars inhibited the growth of the JIMT-1 breast cancer cell line in a dose-dependent manner (Taamalli et al., 2012). An aqueous extract from *Jatropha podagrica* strongly inhibited the growth of both PC12 and A549 in a dose-dependent manner (Ghali et al., 2013). Henna (*Lawsonia inermis*) at doses of 180 mg/kg for 15 days significantly reduced the growth of Dalton's lymphoma ascites-bearing mice (Priya et al., 2011). Treating Ehrlich ascites carcinoma-bearing mice with henna extract decreased the number of cancer cells (Ozaslan et al., 2009). Similarly, the chloroform extract of *L. inermis* was very cytotoxic toward HepG2 (IC_50_ = 0.3 μg/mL) and MCF-7 (IC_50_ = 24.8 μg/mL; Endrini et al., 2002, 2007). Ethyl acetate and petroleum ether extracts both decreased viability of MCF7 cells (IC_50_ = 27 and 22 mg/mL, respectively; El Babili, 2013). The essential oil of henna also showed potent cytotoxicity toward HepG2 (IC_50_ = 24 μg/mL; Rahmat et al., 2006). An aqueous extract of *Urginea maritima* inhibited the growth of SH-SY5Y in a dose- and time-dependent manner (IC_50_ = 10 μg/mL, 1 μg/mL and 100 ng/mL after 24, 48, and 72 h; Elghuol et al., 2016). Both aqueous and ethanol extracts from leaves, seeds and roots of *Plectranthus amboinicus* similarly inhibited the growth of two cancer cell lines, HepG2 and MCF-7 (El-hawary et al., 2012). Hexane, ethyl acetate and chloroform extracts from *Aristolochia baetica* significantly inhibited the growth of MCF-7 cells in a dose- and time-dependent manner (Chaouki et al., 2010). Similarly, ethyl acetate and methanol extracts of *Origanum compactum* both inhibited the proliferation in dose- and time-dependent manner.

## Conclusion

The extensive research on the anticancer activity of plants from the North Africa highlighted their potential for future chemotherapeutic agents, especially plants from Morocco and Algeria, which have the most ethnobotanical references regarding the anticancer effects of plants. Amongst all the botanical families ascribed, Lamiaceae, Apiaceae, Compositae, and Fabaceae are the ones with more plants used in folk medicine. In fact, the most used plants, *T. foenum-graecum, A. longa, M. vulgare*, and *C. absus* belongs to these families. On the other hand, *N. sativa* (Ranunculaceae) is also extensively used as herbal anticancer drug in North African traditional medicine. The anticancer effects of *N. sativa* and *T. foenum-graecum* are widely described both in cytotoxic assays and in mechanisms underlying those effects. Although *A. longa* and *M. vulgare* are referred by herbalists for the treatment of cancer, very few studies assessing their anticancer properties have been conducted while studies demonstrating the anticancer effects of *C. absus* are yet to be conducted. The anticancer effect of these plants act on a panoply of cellular pathways being the induction of apoptosis and modulation of signal transduction pathways the most studied, while the ability to suppress invasiveness, migration and metastasis is poorly evaluated. Although, the safety profile of the plants has been described with minor side effects, *T. foenum-graecum* have been associated with anti-fertility and abortifacient effects and *A. longa* have been described as inducing severe side effects after chronic consumption of the plant. Despite the myriad of studies regarding the anticancer effect as well as extensive literature on the underlying mechanisms, very few *in vivo* studies have been conducted and clinical trials are still to be conducted.

In order to establish these plants as potential chemotherapeutic agents several points must be addressed namely the assessment of anticancer potential using *in vivo* models. Furthermore, clinical trials are still lacking, which limits the therapeutic application of these plants. In addition, a better understanding on the mechanisms of action is still needed in order to establish rational phytotherapeutic approaches.

## Author contributions

JA analyzed the relevant literature, wrote the manuscript and prepared tables and figures. AR, TE reviewed the manuscript. LS supervised the work and review the manuscript.

### Conflict of interest statement

The authors declare that the research was conducted in the absence of any commercial or financial relationships that could be construed as a potential conflict of interest.

## Footnotes

^1^Observatory, G. H. Cancer, deaths per 100,000 Data by country. Available online at: http://apps.who.int/gho/data/node.main.A864?lang=en (Accessed December 17, 2016).
